# *Aux/IAA11* Is Required for UV-AB Tolerance and Auxin Sensing in *Arabidopsis thaliana*

**DOI:** 10.3390/ijms232113386

**Published:** 2022-11-02

**Authors:** Jakub Mielecki, Piotr Gawroński, Stanisław Karpiński

**Affiliations:** Department of Plant Genetics, Breeding and Biotechnology, Institute of Biology, Warsaw University of Life Sciences, 02-787 Warsaw, Poland

**Keywords:** abiotic stress response, hormonal regulation, reactive oxygen species, cell death

## Abstract

In order to survive, plants have, over the course of their evolution, developed sophisticated acclimation and defense strategies governed by complex molecular and physiological, and cellular and extracellular, signaling pathways. They are also able to respond to various stimuli in the form of tropisms; for example, phototropism or gravitropism. All of these retrograde and anterograde signaling pathways are controlled and regulated by waves of reactive oxygen species (ROS), electrical signals, calcium, and hormones, e.g., auxins. Auxins are key phytohormones involved in the regulation of plant growth and development. Acclimation responses, which include programmed cell death induction, require precise auxin perception. However, our knowledge of these pathways is limited. The Aux/IAA family of transcriptional corepressors inhibits the growth of the plant under stress conditions, in order to maintain the balance between development and acclimation responses. In this work, we demonstrate the *Aux/IAA11* involvement in auxin sensing, survival, and acclimation to UV-AB, and in carrying out photosynthesis under inhibitory conditions. The tested *iaa11* mutants were more susceptible to UV-AB, photosynthetic electron transport (PET) inhibitor, and synthetic endogenous auxin. Among the tested conditions, *Aux/IAA11* was not repressed by excess light stress, exclusively among its phylogenetic clade. Repression of transcription by Aux/IAA11 could be important for the inhibition of ROS formation or efficiency of ROS scavenging. We also hypothesize that the demonstrated differences in the subcellular localization of the two Aux/IAA11 protein variants might indicate their regulation by alternative splicing. Our results suggest that *Aux/IAA11* plays a specific role in chloroplast retrograde signaling, since it is not repressed by high (excess) light stress, exclusively among its phylogenetic clade.

## 1. Introduction

The regulation of nuclear gene expression (NGE) by factors and signals originating from organelles is called retrograde signaling [1]. Retrograde and anterograde signaling pathways allow plant cells to receive feedback from their organelles, in order to optimally respond to the surrounding environment and induce a proper response through changes in nuclear gene expression. Understanding how signals derived from organelles, under stress conditions or during biogenesis, affect NGE might be crucial to overcoming current agricultural challenges; for example, water deficits, extreme temperatures, or climate changes [2]. With only a few well-known retrograde pathways, we are still far from understanding the complexity of this regulation, and the precise role of many of the associated factors is yet to be determined. For example, the molecular function of the GENOMES UNCOUPLED1 (GUN1) protein, for which the mutant was isolated nearly 30 years ago, is still unclear [3]. However, plants rely on organellar signaling to survive and adapt to rapid environmental changes. Precise manipulation of these pathways might positively affect the growth and development of plants and establish new strategies to improve their defense reactions. Recently, the participation of many phytohormones in retrograde signaling has been evaluated [4].

Auxins are key hormones that regulate the growth and development of plants. The perceptiveness to gravity, light sources, and induction of adequate tropism movements are all regulated by these phytohormones [5]. Auxins can also integrate various stress signals and coordinate a proper response, which is often connected to adjusting plant growth in order to survive [6]. Auxins are mostly biosynthesized in cotyledons, shoots, and roots, but are present in all plant organs [7]. They are transported through the phloem, in order to distribute this phytohormone throughout the plant organism [8]. Transport over short cell-to-cell distances is carried out by Pin-formed (PIN) proteins [9]. PINs belong to the family of transmembrane proteins that actively contribute to polar auxin transport [10].

Auxins are a group of small molecules that include naturally occurring compounds in plants, such as indole-3-acetic acid (IAA) and phenylacetic acid (PAA), but also synthetic compounds such as 2,4-dichlorophenoxyacetic acid (2,4-D) and 1-naphthaleneacetic acid (NAA). Hormone precursors are also important for auxin signaling pathways, and often referred to as storage forms, such as indole-3-butyric acid (IBA). IBA can be transported through the plant in a conjugated form similar to IAA. During this process, they are bound to protein carriers such as the Transporter of IBA1 (TOB1). It is noteworthy that the conjugated form of auxins is biologically inactive [7]. This form can be stored and eventually converted to IAA through β-oxidation in peroxisomes [11]. Auxins induce the expression of several early auxin response gene families in plants, such as *Auxin/Indole-3-Acetc Acid* (*Aux/IAA*), *Auxin responsive factors* (*ARFs*), and *Gretchen Hagen3* (*GH3*), as well as *Glutathione-S-transferase* (*GH2/4-like*), *Small auxin upregulated RNA* (*SAUR*), and *Aminocyclopropane-1-carboxylic acid synthase* (*ACS*) genes [12].

The *Aux/IAA* family in *Arabidopsis thaliana* encodes 29 short-lived and, in most cases, nuclear proteins that act as repressors of ARFs [13]. The transcriptional regulation mediated by auxin strictly depends on the function of Aux/IAA proteins [14]. Aux/IAA, together with Transport Inhibitor Response 1 (TIR1) or Auxin signaling F-BOX proteins (AFBs), form a co-receptor crucial for auxin sensing [15]. SKP1-cullin-F-box (SCF) ubiquitin E3 ligase forms a complex with TIR1/AFBs to mark the Aux/IAA protein, bound by a auxin molecule, for degradation and to eventually release the ARFs that were bound to the PB1 domain [16]. Apart from TIR1 and ARFs, Aux/IAA proteins also interact with Topless (TPL) transcriptional corepressors through their domain I [17]. Domain I contains an ethylene responsive factor (ERF)-associated amphiphilic repression (EAR) motif, “LxLxL” [12,18]. TPL interaction with histone deacetylase catalyzes the removal of histones acetyl groups, eventually leading to transcriptional repression through DNA condensation [14,17]. Auxin susceptibility, degradation rate, and affinity to different auxins is diverse among the Aux/IAA protein family [14,19,20]. In addition, the promoter sequences of *Aux*/*IAA* family genes contain many different *cis*-regulatory elements governing their expression [21]. These can differ, even among the same phylogenetic clade. This complexity indicates that the function and regulation of Aux/IAA family members might be extremally different, but it also reflects the plurality of metabolic, developmental and acclimation processes that the auxin takes part in.

Aux/IAA proteins are required for stress tolerance and plant survival. *Aux/IAA5* and *Aux/IAA19* regulate resistance to desiccation, and their expression is controlled by C-repeat-binding factor 1 (CBF1) and Dehydration-responsive element-binding protein 2A (DREB2A) [21]. Under stress conditions, both of these proteins bind to the conserved *cis*-regulatory element DRE/CRT, in order to induce *Aux/IAA5* and *Aux/IAA19* expression [21]. *Aux/IAA5, Aux/IAA6,* and *Aux/IAA19* mediate drought tolerance in *Arabidopsis thaliana,* by regulating glucosinolate levels [22]. *OsIAA18* is involved in drought and salt stress tolerance in rice [23]. *Aux/IAA14* regulates cold stress response in Arabidopsis roots [24]. *Aux/IAA17* confers salt stress resistance in *Arabidopsis thaliana* [25]. In contrast to these loss-of-function mutants, several gain-of-function, auxin-resistant mutants have been described in the literature. This type of mutation affects auxin’s ability to degrade *Aux/IAA* proteins, eventually making the plants auxin-resistant. The phenotypes of these gain-of-function mutants match transgenic lines with *Aux/IAA* overexpression [26,27]. As an example, the *auxin resistant 2* (*axr2*) mutant in the *Aux/IAA7* gene exhibits a dwarf phenotype and stunted growth [28], while *auxin resistant 5* (*axr5*), with a mutation in the *Aux/IAA1* gene, produces defective root and shoot tropism movements [29]. Apart from abiotic stresses, *Aux/IAA* also take part in biotic defense responses [30,31] and fruit ripening [32]. Heat shock protein 90 (HSP90) stabilizes the auxin coreceptor in stressful conditions, allowing Aux/IAA degradation and the expression of genes regulated by the released ARFs [33].

The abnormal phototropism in *Aux/IAA* mutants indicates the role of Aux/IAA proteins in the cross-talk between hormones and light sensing. Aux/IAA3 represses Phytochrome-Interacting-Factors (PIFs) through a physical interaction between domain I of Aux/IAA and PIFs [34]. PIFs are a subfamily of basic helix-loop-helix (bHLH) transcription factors (TFs) negatively regulating plant photomorphogenesis. Aux/IAA7, Aux/IAA12, and Aux/IAA17 can physically interact with photoreceptors, including phytochromeB (phyB), playing a major role in red and far-red light perception and Cryptochrome1 (CRY1), a key blue light receptor [35]. Both of these interactions prevent Aux/IAA degradation through TIR1 and block expression of ARFs related genes after illumination [35].

Recent works have also presented the interaction between auxins and chloroplast-, and mitochondria-to-nucleus retrograde signaling. One of the metabolites synthetized in chloroplasts and involved in chloroplast-to-nucleus communication, methylerythritol cyclodiphosphate (MEcPP), coordinates hormonal and light signals through phyB and PIFs accumulation [36]. As such, MEcPP reduces the abundance of PIN1, an auxin transporter located in the plasma membrane that affects auxin polar transport [37]. The exact role of auxins in chloroplast retrograde signaling is yet to be determined; however, growing evidence suggests that this hormone can also interact with another chloroplast-to-nucleus signaling pathway, SAL1-PAP. In accordance with this, it was observed that the lack of SAL1 phosphatase in *sal1* mutant increases the cellular auxin level [38,39]. Auxins are also crucial in mitochondrial retrograde signaling. It was observed that induction of mitochondrial stress results in transient suppression of auxin signaling [1,40]. On the other hand, perturbation of auxin transport and biosynthesis induced expression of mitochondrial retrograde signaling markers [41,42]. Thus, it is suggested that auxin signaling and mitochondrial retrograde signaling operate antagonistically [41,42].

Although, many findings have identified auxins as playing a role in photosynthesis, plastid biogenesis, mitochondrial metabolism, and retrograde signaling [43,44], the full picture of this process remains unclear. Thus, the presented work aimed to expand on existing knowledge about auxins’ involvement in chloroplasts-to-nucleus retrograde signaling. We performed a reverse genetic screen for potential retrograde signaling-related TFs using chloroplast targeted stresses. Recently, this strategy has allowed us to identify novel putative transcriptional and translational, nuclear and chloroplast regulators, respectively [45]. Based on this screen, we focused on the functional characterization of *Aux/IAA11* (AT4G28640) gene and its involvement in the response to abiotic stresses. Bioinformatic analysis of *Arabidopsis thaliana* Aux/IAA proteins using the Aramemnon [46] plant membrane protein database predicted that *Aux/IAA11* is the only member encoding the potential transmembrane region among its family members [46,47]. In summary, our results demonstrated that Aux/IAA11 is involved in auxin sensing and abiotic stress tolerance in *Arabidopsis*.

## 2. Results

### 2.1. Reverse Genetic Screen for Membrane Bound Transcription Factors Involved in Retrograde Signaling

To expand our knowledge on chloroplast-to-nucleus retrograde signaling pathways, we performed a reverse genetic screen using T-DNA mutant lines in nine different genes that encode potential TFs containing transmembrane (TM) regions. Candidate genes were selected based on the presence of TMs and on the mining of transcriptomic data of plants exposed to chloroplast-specific stresses using Genevestigator [48]. Selection criteria required at least a twofold increase or decrease of gene expression in response to light stresses, light quantity changes, or PET inhibitors. All tested mutants were obtained from Nottingham Arabidopsis Stock Centre (NASC, United Kingdom) [49] in Columbia (Col-0) ecotype background. Our aim was to find new membrane bound TFs that, similarly to PTM [50], after exposure to stress could lead to its detachment from the chloroplast envelope and eventually change the nuclear gene expression, in order to cope with stress conditions. The mutants used in this screen are listed in Table S1.

To test for potential involvement of the analyzed genes in chloroplast-to-nucleus retrograde signaling, we exposed T-DNA mutants to three different chloroplast-targeted photooxidative stresses: UV-AB radiation, 3-(3,4-dichlorophenyl)-1,1-dimethylurea (DCMU), and methyl viologen (MV). This strategy had previously been shown to be successful and led to the identification of CIA2 and CIL [45]. Disruption of the communication between chloroplasts and nucleus, due to the introduced T-DNA mutation in the tested lines, should lead to deregulated susceptibility to those abiotic stresses if those mutated factors are involved in retrograde signaling pathways. Ultraviolet radiation in small doses may be beneficial to plants [51]. However, absorption of high amounts of UV leads to abnormal growth and development. It affects the synthesis and replication of DNA, by forming dimers between pyrimidines [52]. UV radiation also induces oxidative stress through ROS formation, leading to oxidation of proteins and lipids [53]. The main target of UV at the cellular level is the chloroplast, and this causes photoinhibition, affecting the maximum quantum yield of photosystem II (*F_v_*/*F_m_*) [54]. Eventually, this stress can lead to the induction of programmed cell death (PCD) [55]. Apart from UV-AB, plants were also treated with the photosynthesis inhibitors DCMU and MV, in order to induce ROS production and photoinhibition. DCMU specifically and noncovalently binds to the quinone B binding site on the D1 protein, thereby inhibiting photosynthetic electron transport between quinone B and plastoquinone, causing overreduction of quinone A and oxidation of the plastoquinone pool [56,57]. This leads to the formation of a singlet oxygen in PSII [58]. MV in chloroplasts competes with ferredoxin for electrons on the photosystem I (PSI) acceptor site, eventually leading to superoxide ($O_{2}^{-}$^●^) formation. Subsequently, superoxide can form other ROS and lead to cell death [59].

All tested T-DNA mutant lines exhibited phenotypes similar to the wild-type under control ambient conditions (Figure S1). Thus, to test their involvement in chloroplast-to-nucleus retrograde signaling, we exposed the T-DNA mutants to UV-AB, DCMU, and MV. To analyze the UV-AB susceptibility, we exposed plants to this radiation and after 72 h measured the ion leakage, as a proxy for cell death, from whole plant rosettes. After the exposure to UV-AB radiation, we observed that three T-DNA mutants (SALK_090823 (AT3G47550, *F1P2.100*), SALK_140301 (AT5G05660, *NFXL2*), and GK-685A08 (AT4G28640, *Aux*/*IAA11*)) were more susceptible than wild type (Col-0) plants to this stress (Figure S2). Additionally, we exposed leaf-disks to two potent photosynthesis inhibitors (DCMU and MV) and analyzed *F_v_*/*F_m_* as a measure of photoinhibition. Based on these experiments, we observed that only GK-685A08 (AT4G28640, *Aux*/*IAA11*) exhibited a higher susceptibility to DCMU, and we did not observe an altered susceptibility to MV among all genotypes tested (Figure S2).

**Figure 1:**
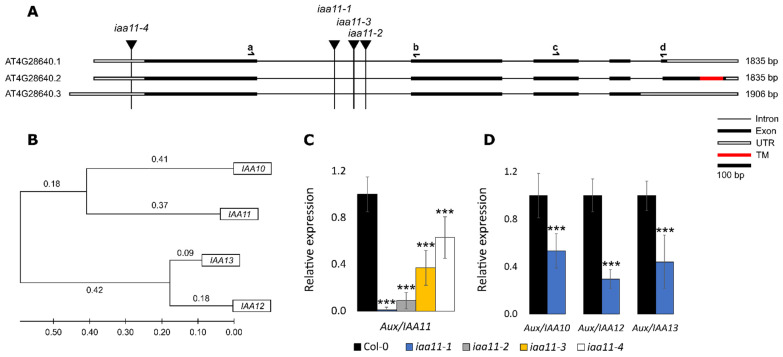
Identification of T-DNA mutants in *Aux/IAA11* gene, and phylogenetic analysis of Aux/IAAs. (**A**) Splicing variants of *Aux/IAA11* gene. Black vertical lines represent the insertion of T-DNA in *iaa11-1*, *iaa11-2*, *iaa11-3*, and *iaa11-4* mutant lines. The red rectangle indicates the localization of the transmembrane (TM) domain in *Aux/IAA11.2.* Black half arrows illustrate binding sites of the primers used for qPCR. (**B**) Cladogram of protein similarities. *Aux/IAA11* clade was prepared using the ClustalW alignment algorithm and the maximum likelihood statistical method in Molecular Evolutionary Genetics Analysis (MEGA) 11 software [60]. (**C**) Relative expression of *Aux/IAA11* in tested mutant lines. (**D**) Relative expression of *Aux/IAA10*, *Aux/IAA12*, and *Aux/IAA13* in *iaa11-1* mutant background. Relative gene expressions were calculated and normalized with *UBIQUITIN-PROTEIN LIGASE 7 (UPL7*) and *YELLOW-LEAF-SPECIFIC GENE 8* (*YLS8*) as references, using the Relative Expression Software Tool (REST 2009) (n = 6, *** *p* < 0.001). Error bars represent the standard deviation.

As a phenotype of increased susceptibility to two chloroplast-specific stresses (UV-AB and DCMU) in the tested AT4G28640, the *Aux/IAA11* mutant focused our efforts on further characterizing this gene. Thus, to evaluate if the observed reverse genetic screen results were related to disruption of the Aux/IAA11 gene, we ordered another three T-DNA mutant alleles in the Aux/IAA11 gene from NASC (United Kingdom). In addition to the mutant initially analyzed (GK-685A08 (*iaa11-1*)), we included the following mutant alleles: SAIL_56_H06.1 (*iaa11-2*), SALK_115462 (*iaa11-3*), and SALK_033787 (*iaa11-4*). All T-DNA lines were genotyped and we also sequenced the PCR products obtained using T-DNA and gene-specific primers, to check the exact integration site of T-DNA in the *Aux*/*IAA11* gene sequence (Figure 1A). Every tested line, except SALK_033787, had a T-DNA insertion in the first intron of the *Aux*/*IAA11* gene. *iaa11-1*, *iaa11-2*, and *iaa11-3* have T-DNA inserted 560, 652, and 617 bp after the translation start site (TSS), respectively. The *iaa11-4* has a T-DNA insertion located 41 bp before TSS, in the 5′ UTR region.

### 2.2. Expression of Aux/IAA11 Gene in the Tested Lines and Expression Patterns

To evaluate if insertion of T-DNAs into *the Aux/IAA11* gene affected its expression, we performed quantitative PCR (qPCR) using primers (Table S2) that flank the first intron of the *Aux/IAA11* gene. Each tested mutant line exhibited significantly lower expression of the *Aux/IAA11* gene than the wild type. *iaa11-1* only had residual expression (1% of WT), while the other mutants with T-DNA introduced into the first intron had 9–37% of the wild-type expression in *iaa11-2* and *iaa11-3,* respectively (Figure 1C). *iaa11-4* with T-DNA inserted into the 5′UTR sequence exhibited a higher expression, around 63%, than any other mutant line. However, this is still 37% lower than the expression measured in wild-type plants. We also checked if knock-out of *Aux/IAA11* in the *iaa11-1* mutant was compensated by a higher expression of other *Aux/IAA* family members from the same clade (Figure 1B). Surprisingly, we discovered that the expressions of *Aux/IAA10, Aux/IAA12,* and *Aux/IAA13* were, respectively, 47%, 71%, and 66% lower than in wild-type plants without any treatment (Figure 1D). These results suggest that the integration of T-DNA into the analyzed mutants significantly affected the expression of *Aux/IAA11* and other *Aux/IAA* genes of the same family, suggesting that Aux/IAA11 can influence the expression of these genes.

**Figure 2:**
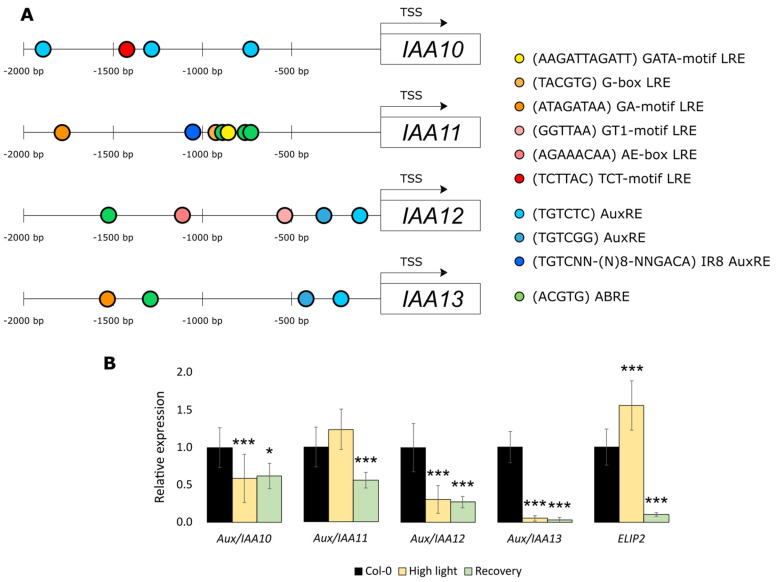
Promoter analysis and light induction of the *Aux/IAA11* genetic clade. (**A**) Location of light responsive elements (LREs), auxin responsive elements (AuxREs), and abscisic acid responsive elements (ABREs) in promoter sequences 2000 bp upstream from the translation start site (TSS) of *Aux/IAA10*, *Aux/IAA11*, *Aux/IAA12*, and *Aux/IAA13* genes. (**B**) Expression of the *Aux/IAA11* phylogenetic clade in response to high light stress. Relative gene expressions were calculated and normalized with *UBIQUITIN-PROTEIN LIGASE 7* (*UPL7*) and *YELLOW-LEAF-SPECIFIC GENE 8* (*YLS8*) as references, using Relative Expression Software Tool (REST 2009) (n = 6, * *p* < 0.05; *** *p* < 0.001). Error bars represent the standard deviation.

Connection of the expression of *Aux/IAA10*, *Aux/IAA12*, and *Aux/IAA13* with *Aux/IAA11* in the *iaa11-1* mutant led us to analyze the structure of promoters in this gene family. Members of the *Aux/IAA* family are generally classified as early auxin response genes [61]. Promoters of the *Aux/IAA10*, *Aux/IAA12*, and *Aux/IAA13* genes have one or both canonical auxin responsive elements (AuxRE): TGTCTC and TGTCGG (Figure 2A). In contrast, the promoter of the *Aux/IAA11* gene does not contain either of these two types of AuxRE. However, there is one novel AuxRE called IR8 and located around 1000 bp upstream from start of translation site. Induction of *Aux/IAA11* gene with auxin through the IR8 element was recently described by Freire-Rios et al. [62]. Promoter sequences of all genes from this genetic clade, except *Aux/IAA10*, also contain at least one ACGTG abscisic acid responsive element (ABRE). These elements can bind to the basic leucine zipper (bZIP) transcription factors that regulate plant development and resistance to abiotic stresses [63]. Promoters of *Aux/IAA12* and *Aux/IAA13* have only one ABRE *cis*-regulatory element, while *Aux/IAA11* have three of those elements, repeated closely after each other. In the promoter sequence of *Aux/IAA10* gene, we found only one light-responsive element (LRE), TCT-motif (TCTTAC), located 1426 bp upstream from the start of translation. Another two different types of LREs were found in the *Aux/IAA12* promoter AE-box (AGAAACAA) and GT1-motif (GGTTAA) located, respectively, 963 and 315 bp upstream from the translation start. In the promoter sequence of *Aux/IAA13* gene, we found the GA-motif (ATAGTTAA) located 1214 bp upstream from translation start. The same GA-motif type LRE was found in the *Aux/IAA11* gene promoter located 1617 bp upstream from start of translation. Additionally, in the promoter sequence of the *Aux/IAA11* gene, we found another two types of LREs that were absent in the other analyzed *Aux/IAA* promoters from this phylogenetic clade: G-box (TACGTG) and GATA-motif (AAGATAAGATT), located, respectively, 775 and 665 bp upstream from the start of translation. These results suggest a different regulation of *Aux/IAA11* expression through auxins, ABA, and light than in other genes from the same phylogenetic clade. Thus, we wanted to check if *Aux/IAA11* and other similar genes from the same phylogenetic clade (Figure 1B) were regulated in response to high light stress. We measured the expression of AT1G04100 (*Aux/IAA10*), AT4G28640 (*Aux/IAA11*), AT1G04550 (*Aux/IAA12*), and AT2G33310 (*Aux/IAA13*) in Col-0 after excess light stress using qPCR (Figure 2B). As confirmation that the tested plants suffered from excess light induced photooxidative stress in our experimental conditions, we also checked the expression of *Early light-induced protein2* (*ELIP2*) [64]. Surprisingly, *Aux/IAA11* was not only not repressed, but also slightly induced during this treatment. Other genes from the same clade were downregulated, even after 24 h of recovery after stress in ambient laboratory conditions. In summary, we suggest that *Aux/IAA11* has a unique expression profile among *Aux/IAA* genes, probably due to the presence of several abscisic acid responsive (ABREs) and light responsive (LRE) *cis*-regulatory elements in the promoter region 1000 bp upstream of the translation start site (TSS).

### 2.3. Aux/IAA11 Is Involved in UV-AB Stress Tolerance

**Figure 3:**
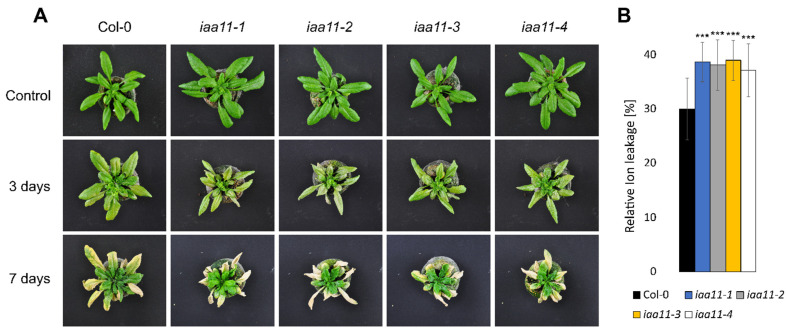
UV-AB susceptibility of *Aux/IAA11* mutants. (**A**) Photographs of tested *Arabidopsis thaliana* plants before, and 3 and 7 days after, the UV stress exposure. (**B**) Relative ion leakage measured 3 days after UV stress exposure. n = 9, Dunnett’s test (*** *p* < 0.001). Error bars represent the standard deviation.

In the initial screen, we identified *Aux/IAA11* as a gene that may be involved in UV-AB stress tolerance, since *iaa11-1* was more susceptible to this irradiation (Table S1, Figure S2). We repeated the UV treatment on all four obtained T-DNA mutants in the *Aux/IAA11* gene. We decided to use a higher dose of UV-AB, 1500 instead of 1000 mJ cm^−2^, because we wanted to impose a stronger effect of UV on the tested plants, to see if this phenotype can also be induced with different doses. All tested T-DNA mutants exhibited a higher susceptibility to UV-AB stress (Figure 3). The ion leakage measured 3 days after stress was approximately 8% higher in *iaa11-1*, *iaa11-2*, *iaa11-3,* and *iaa11-4,* which confirmed the higher cell death in plants lacking the *Aux/IAA11* gene after UV-AB exposure. We observed that higher cell death in mutant lines occurred more often in older, mature leaves, while young leaves seemed to be less affected compared to the wild-type. This indicates the involvement of the *Aux/IAA11* gene in UV-AB stress responses.

### 2.4. Aux/IAA11 Is Required for Carrying Out Photosynthesis under Inhibitory Conditions

**Figure 4:**
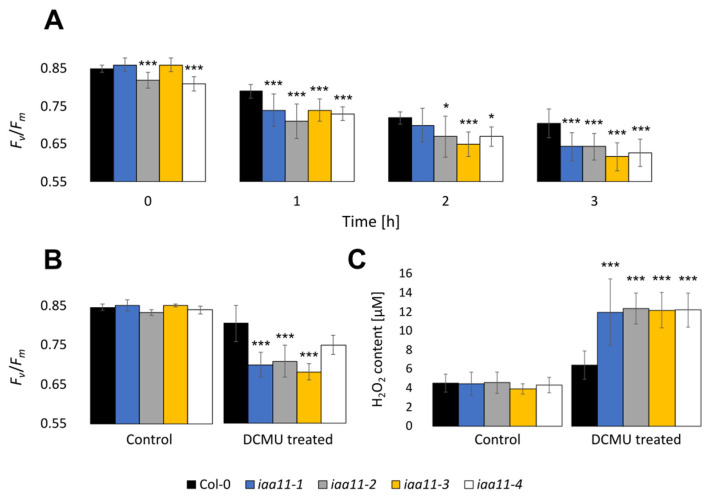
3-(3,4-dichlorophenyl)-1,1-dimethylurea (DCMU) susceptibility of *Aux/IAA11* mutants. (**A**) Maximum quantum efficiency of photosystem II (*F*_v_/*F*_m_) after 0, 1, 2, and 3 h of 60 µM DCMU treatment (n = 9). (**B**) *F*_v_/*F*_m_ and (**C**) H_2_O_2_ content measured after 15 min of 200 µM DCMU treatment. n ≥ 6, Dunnett’s test (* *p* < 0.05; *** *p* < 0.001). Error bars represent the standard deviation.

Similarly to the UV-AB treatment, we also repeated the DCMU treatment on all four tested T-DNA lines with a mutation in the *Aux/IAA11* gene (Figure 4). We observed a lower maximum quantum yield of photosystem II (*F*_v_/*F*_m_) after 1, 2, and 3 h of photosynthesis inhibitor treatment in all tested *iaa11* mutants. After 3 h of treatment, *F*_v_/*F*_m_ values were 0.06–0.09 lower in mutant lines compared to the wild-type plants, which indicated a stronger photoinhibition in *Aux/IAA11* mutants during the DCMU treatment (Figure 4A).

**Figure 5:**
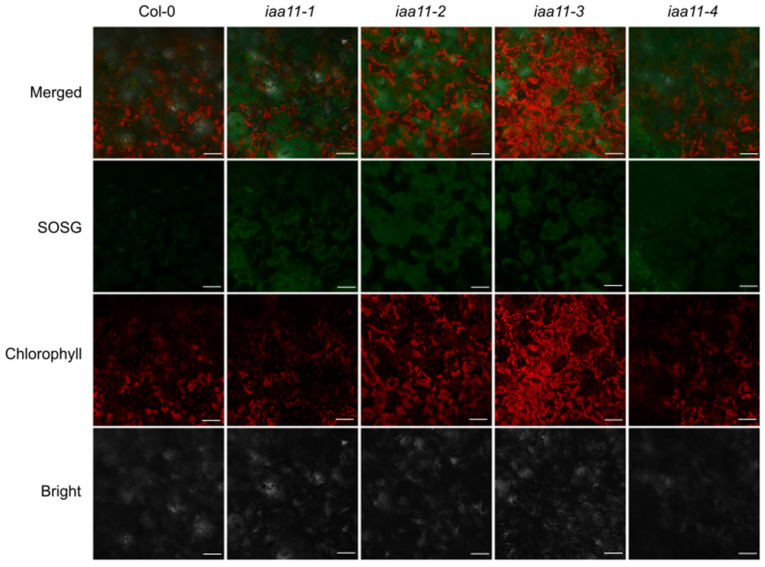
Visualization of singlet oxygen sensor green (SOSG). Images were taken 2 and 5 h after a 15 min treatment with 200 µM DCMU and SOSG staining. Green and red colors represent SOSG and chlorophyll fluorescence, respectively. White scale bars represent 50 µm.

In addition to *F*_v_/*F*_m_, we also measured the content of hydrogen peroxide (H_2_O_2_), which, in contrast to singlet oxygen, has a longer half-time and might be able to relocate between cell compartments and, systemically, between plant cells [2,57,65]. *Aux/IAA11* mutants after stress accumulated higher amounts of H_2_O_2_ than Col-0 after a 200 μM DCMU treatment. The hydrogen peroxide concentration measured in the *Aux/IAA11* mutant lines was approximately 12 μM, while in Col-0 it was almost twice as low, 6 μM of H_2_O_2_ (Figure 4C). Similarly to the previous experiments with lower doses of DCMU, we observed a higher photoinhibition in the tested lines with a mutation in the *Aux/IAA11* gene (Figure 4B). To evaluate the formation of singlet oxygen among the tested T-DNA mutants, we also stained leaf discs, treated with 200 μM of DCMU, with singlet oxygen sensor green (SOSG), as described by Flors et al. [56]. Higher concentrations of singlet oxygen among the tested *Aux/IAA11* mutants were confirmed by visualization of SOSG using confocal microscopy (Figure 5). A higher SOSG fluorescence was observed in all tested *Aux/IAA11* mutants (Figure S4). However, we think that the SOSG signal we observed was a secondary effect related to the formation of singlet oxygen from lipid hydroperoxide (LOOH), rather than the chloroplasts themselves. The Pearson correlation coefficient confirmed no correlation between the SOSG and Chlorophyll *a* fluorescence signals (Figure S4). The excess of singlet oxygen and other ROS produced in the chloroplasts after DCMU treatment can lead to lipid peroxidation, of which LOOHs are primary products. LOOHs can be reduced by glutathione peroxidases (GPx), which eventually generate fatty acid peroxyl (LOO●) and which can be a source of singlet oxygen formation, through the Russell mechanism [66]. Photosynthesis was not affected in the *Aux/IAA11* mutants not treated with DCMU (Figure S3).

### 2.5. Aux/IAA11 Is Involved in Auxin Sensing

In order to evaluate if a lower expression of *Aux/IAA11* affects auxin sensing, we tested mutant lines grown in vitro with or without supplementation with synthetic auxin: 2,4-Dichlorophenoxyacetic acid (2,4-D). We also introduced the *auxinresistant2* (*axr2*) mutant into our experiment, as an additional control, which was expected to be insensitive to 2,4-D. The *axr2* has a single point mutation that leads to a change in the amino acid sequence, GWSP instead of GWPP, in the so-called degron motif in domain II of the mutated Aux/IAA7 protein [28]. The GWPP motif is responsible for auxin molecule sensing and also the formation of a complex with TIR1 protein [67]. In the presence of an auxin molecule, the (SCF)^TIR1/AFBs^ complex is formed, in order to mark Aux/IAA for degradation and release the ARFs that were bound to the PB1 domain. *axr2* cannot form this complex and is almost completely insensitive to auxin; it is described in the literature as a gain-of-function mutant [67]. On the contrary, the lower expression of *Aux/IAA11* in the tested mutant lines should have resulted in a higher auxin susceptibility. The Aux/IAA11 protein, similarly to Aux/IAA10, Aux/IAA12, and Aux/IAA13, also has a conserved GWPP amino acid motif in its sequence, indicating its auxin susceptibility. 2,4-D mimics the effect of auxins in plants. Its high concentration in the roots of auxin-sensitive plants inhibits the main root growth and induces the formation of lateral roots [19]. However, auxin-insensitive plants should not be affected by 2,4-D treatment and their roots should develop undisturbed.

**Figure 6:**
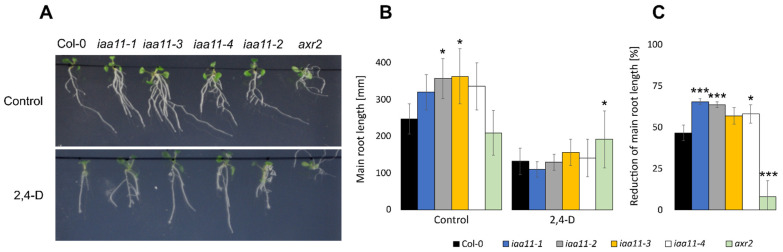
*Aux/IAA11* is involved in auxin sensing. (**A**) Phenotype of 7-day-old *Aux/IAA11* mutants and *auxinresistant2* (*axr2*) after treatment with 2,4-Dichlorophenoxyacetic acid (2,4-D). (**B**) Main root length of plants grown on ½ MS and on ½ MS supplemented with 10 nM of 2,4-D. (**C**) Reduction of the main root length of plants grown on 2,4-D compared to control conditions. n ≥ 6, Dunnet’s test (* *p* < 0.05; *** *p* < 0.001). Root lengths were measured using ImageJ software. Error bars represent standard deviation.

After 7 days of in vitro growth, *iaa11-1*, *iaa11-2*, *iaa11-3*, and *iaa11-4* exhibited longer main roots, as well as more lateral roots in the control without 2,4-D supplementation, but we observed that this only occurred in the early development and did not affect plant growth later on (Figure 6A). Plants treated with 2,4-D, except *axr2*, exhibited a severe reduction in main root length (Figure 6B). Although the *axr2* main root length was reduced on average only by 8%, in Col-0 this was 47%. All the tested *Aux/IAA11* T-DNA mutants were more affected by 2,4-D than the wild type. The main root lengths of the *iaa11-1*, *iaa11-2*, *iaa11-3*, and *iaa11-4* lines were reduced by 65, 64, 57, and 58%, respectively (Figure 6C). In the tested mutant lines, we also observed more root hairs on the lateral roots and main root cap, which is also connected to their higher auxin susceptibility. In summary, these results suggest that the repressor activity of Aux/IAA11 is required for physiological auxin sensing. The lower expression of *Aux/IAA11* in the tested mutants resulted in a higher, deregulated susceptibility to auxin when compared to the wild type and *auxinresistant2*.

### 2.6. Subcellular Localization of Aux/IAA11 Protein Variants

**Figure 7:**
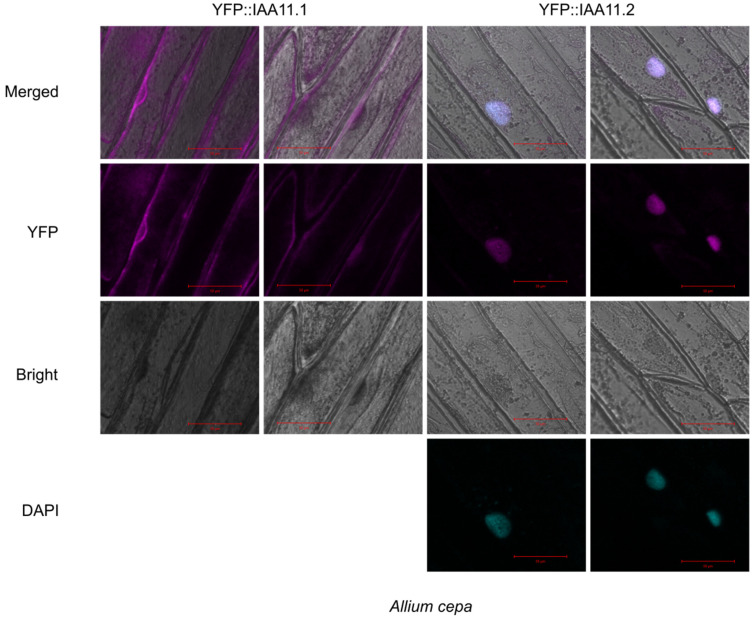
Subcellular localization of IAA11 protein variants in *Allium cepa*. Transient expression of fusion proteins: YFP::IAA11.1 and YFP:IAA11.2 in *Allium cepa* adaxial epidermis under 35S constitutive promoter. Pictures were taken 72 h post agrobacterium infiltration using confocal microscopy. Magenta and cyan colors represent Yellow fluorescent protein (YFP) and 4′,6-Diamidino-2-Phenylindole, Dihydrochloride (DAPI) fluorescence, respectively. Red scale bars represent 50 µm.

Aux/IAA11 is the only protein containing a transmembrane (TM) region among all of the Aux/IAA family members. In order to determine its subcellular localization, we obtained genetic constructs based on two *Aux/IAA11* splicing variants: AT4G28640.1 (IAA11.1) and AT4G28640.2 (IAA11.2). IAA11.1, unlike the canonical protein variant IAA11.2, lacked the predicted transmembrane region near the C-terminus end of the protein (Figure 1A). Thus, to analyze the subcellular localization, both protein variants were fused with Yellow fluorescent protein (YFP) at the N-terminal end. The expression of fusion proteins was driven by 35S constitutive promoter. Both constructs were transiently transformed using *Agrobacterium tumefaciens* C58C1, into adaxial *Allium cepa* epidermis (Figure 7) and *Nicotiana benthamiana* abaxial epidermis (Figure S5). The YFP::IAA11.1 expression was observed in the cytoplasm in both transformed organisms, while YFP::IAA11.2, containing a transmembrane region, was expressed in the nucleus, which was confirmed by 4′,6-Diamidino-2-Phenylindole, Dihydrochloride (DAPI) staining in *Allium cepa* (Figure 7 and Figure S6). In addition, we also observed a low YFP fluorescence in the cytoplasm of tobacco plants transformed with this construct. In summary, we suggest that the subcellular location of both variants is different, with IAA11.1 and IAA11.2 localized in the cytoplasm and nucleus, respectively.

## 3. Discussion

Growing evidence suggests an interplay between auxin and light, and chloroplast-to-nucleus retrograde signaling [35]. Within this work, we identified a new protein, Aux/IAA11, which can participate in chloroplast-to-nucleus signaling and integrate this pathway with auxin signaling. As such, we observed that disruption of the *Aux/IAA11* gene leads to an increased susceptibility to chloroplast specific stresses (UV-AB and DCMU) and auxin treatment. These results suggest that Aux/IAA11, as a repressor of ARF, could be important during chloroplast dysfunction, to restrict growth and redistribute resources to effectively acclimate to stress. The interplay of Aux/IAA11 with other Aux/IAAs may be important in this process. Previous works suggested that the Aux/IAA network is complex [12,21], and the exact role of Aux/IAA11 needs to be investigated.

However, our in silico analysis of gene promoters suggested that the different AuxREs in *Aux/IAA10*, *Aux/IAA11*, *Aux/IAA12*, and *Aux/IAA13* might be regulated by different ARFs (Figure 2A). Novel AuxRE, IR8 located in the promoter of *Aux/IAA11*, can be bound by ARF5 and ARF1 [62]. However, the nucleotide sequence of AuxRE might not be the only factor determining the specificity of binding by ARFs [68]. More important might be how many AuxREs are repeated after each other in a close vicinity. The IR8 motif found in the promoter of *Aux/IAA11* consists of an inverted repeat (TGTCNN) separated by a 8 bp spacer. Importantly, inverted repeats more efficiently bind ARFs than single repeats or direct repeats [62,68], which might suggest a tight regulation of *Aux/IAA11* by ARFs. In addition to IR8, there are three ABREs located in the promoter of *Aux/IAA11*. ABREs can be bound by different bZIPs, in response to osmotic stress, cold, drought, or ABA treatment [69,70]. It was previously shown that promoters of several *Aux/IAA* are regulated by one of the best-characterized bZIPs, DREB2A [21]. We observed that among all genes of the *Aux/IAA11* phylogenetic clade, the promoter of *Aux/IAA11* was the most enriched in ABREs compared to the other gene promoters (Figure 2A). This supports the previous observation that the expression of *Aux/IAA11* is induced after 30 min of desiccation stress [21]. This might suggest a significant *Aux/IAA11* regulation by ABA, which is consistent with the role of ABA in chloroplast stress signaling [71,72,73]. *Ascorbate peroxidase 2* (*APX2*) gene, similarly to *ELIP2*, is induced by excess light stress. *APX2* encodes the hydrogen peroxide scavenging enzyme important in the response to the oxidative stress. *APX2* expression is also regulated by ABA and chloroplast retrograde signaling [57,74,75].

*Aux/IAA11* is also exclusively induced by high light stress, among its phylogenetic clade (Figure 2). Convergent results were recently presented by Huang et al. [76], where transcriptome profiling using RNA sequencing was performed after constant high-light stress. Most of the RNAs that encode Aux/IAA proteins were underrepresented compared to the control group in this experiment. In our study, we tried to replicate a similar experimental setup, and we can conclude that *Aux/IAA11* might be the only gene that is not repressed, and even slightly induced, by high light among its phylogenetic clade and maybe the entire family. This might suggest a specific role of Aux/IAA11 when compared to the other family members. The abundancy of different light responsive cis-regulatory elements in the promoter of *Aux/IAA11* might partially explain the higher induction of this gene by light than any other member of its phylogenetic clade.

The presented results suggest the involvement of *Aux/IAA11* in UV-AB stress response (Figure 3). Interestingly, the UV-B photoreceptor UVR8 regulates auxin responses and lateral root development through interaction with MYB73/MYB77 transcription factors [77]. MYB77 interacts with ARFs, affecting transduction of auxin signaling and eventually lateral root growth [78]. MYB77 also upregulates the expression of *Aux/IAA19* and *Aux/IAA1* binding to the promoter through one or two types of MYB binding motif: MRE (AACC or TTGG) and/or MYBcore (CAGTTG or CAACGG) cis-regulatory elements [79]. Two CAACGG MYBcore motifs are present in the *Aux/IAA11* promoter region but not in other genes from the same phylogenetic clade (Figure 2). However, the promoter of *Aux/IAA13* contains the CAGTTG MYBcore motif. We also observed that all genes among this phylogenetic clade contain several MRE motifs. The involvement of Aux/IAA11 in UV-AB resistance through MYB77/UVR8 regulation requires further investigation. Additionally, it was observed that UV-B stabilizes Aux/IAA proteins [80]. In this work, we observed that plants with mutation in *Aux/IAA11* were unable to inhibit their growth in response to UV-AB and were more susceptible to this stress factor, accompanied by increased PCD (Figure 3). Inhibition of growth through auxins is one of the strategies to cope with abiotic stress [81], to effectively acclimate to stress conditions, and we suggest that Aux/IAA11 might participate in this process. Under stressful conditions, when cells can no longer can maintain metabolic processes, they cross a certain threshold and induce PCD [82]. *Aux/IAA5* expression is lower in the *lesion simulating disease 1* (*lsd1*) mutant background [83]. This might indicate the involvement of Aux/IAA5 in LSD1-dependent cell death regulation. *Aux/IAA11*, similarly to *LSD1*, is a negative cell death regulator. However, their cooperation or direct regulation is yet to be determined. The cell cycle under UV stress is inhibited, in order to prevent introduction of mutations in the DNA during replication [84]. Recently, more evidence for the convergence of light and auxin signaling pathways was presented in the literature [85]. Collectively, these data indicate that Aux/IAA11 may be involved in the UV-AB stress response.

UV-AB and HL are factors that significantly influence chloroplast function. To better understand the role of Aux/IAA11 in chloroplast stress, we exposed *iaa11* mutant plants to the potent PET inhibitor DCMU (Figure 4). Interestingly, we observed that after DCMU treatment, disruption of *Aux/IAA11* led to an increased inhibition of PSII, and the mutant plants exhibited higher susceptibility to this herbicide (Figure 4). Higher photoinhibition eventually led to higher ROS formation (Figure 4C and Figure 5). However, it would be interesting to evaluate if this is related to a lower singlet oxygen production by PSII or a higher activity of ROS scavenging enzymes when *the Aux/IAA11* gene is functional. There is also another link between oxidative stress and auxin, since the formation of IAA from IBA requires β-oxidation [11]. Moreover, spraying plants with IAA during senescence also affects the maximum efficiency of PSII (*F_v_*/*F_m_*), showing a correlation between cellular auxin level and PSII efficiency [86]. In summary, the induction of *Aux/IAA11*, and increased susceptibility to chloroplast-targeted stresses, suggest that *Aux/IAA11* is important for proper acclimatory responses, likely through its role in the suppression of auxin signaling.

Auxin signaling pathways contain several negative feedback loops, for example Aux/IAA proteins are able to autorepress their own genes through the release of ARFs [87]. Another layer of complexity is added by the fact that one ARF, upon release from Aux/IAA, can regulate the expression of many different *Aux/IAA* genes [12]. Moreover, Aux/IAAs can compete with each other to bind to the ARFs [88]. Such an interdependence of Aux/IAAs is observed in a case of *Aux/IAA33*, where the disruption of this gene in the *iaa33* mutant background is compensated by the increased expression of other *Aux/IAAs* [88]. Notably, Aux/IAA33 is accumulated in the presence of auxins, instead of degraded, which might indicate a different regulation and function of this non-canonical Aux/IAA protein [88].

In our experimental setup, we also observed that the expression of *Aux/IAAs* depended on the expression of the *Aux/IAA11* gene. The decreased expression of *Aux/IAA11* in *iaa11-1* mutants (Figure 1C) leads to a lower expression of *Aux/IAA10*, *Aux/IAA12*, and *Aux/IAA13* (Figure 1D), and to a higher 2,4-D susceptibility (Figure 6). This might indicate that Aux/IAAs are deregulated through, even partially, decreased expression of *Aux/IAA11*. Such deregulation was also observed in *axr2* mutant, where a point mutation in just one Aux/IAA protein results in auxin resistance and the reduction of growth and development [28]. We also observed that the *axr2* auxin-resistant mutant was barely affected by 2,4-D (Figure 6). Collectively, these data suggest that the phenotype we observed is strictly related to 2,4-D supplementation and the higher susceptibility of *Aux/IAA11* mutants.

The *Aux/IAA11* gene in the present annotation of *A. thaliana* genome has three isoforms. All isoforms contain the same conserved domains (I, II, III, and IV) characteristic of canonical Aux/IAA proteins. The first three exons of each isoform are identical, alterations in splicing of intron 4, leading to different protein variants (Figure 1). Only Aux/IAA11.2 (canonical protein variant) poses a putative TM domain at the C-termini, suggesting that it can be anchored to the membranes. This observation led us to analyze the subcellular localization of Aux/IAA11.1 and Aux/IAA11.2 fused to the fluorescent tag in vivo, using two transient transformation systems (Figure 7 and Figure S5). We observed that the canonical protein variant (Aux/IAA11.2) containing the transmembrane region localizes in the nucleus (Figure 7 and Figure S5) and does not appear to be anchored to any membrane. These results confirmed the observations that Aux/IAAs are nuclear proteins [35]. Surprisingly, Aux/IAA11.1, unlike other Aux/IAAs, localizes in the cytoplasm, which is unexpected, since the known Aux/IAA interaction partners e.g., TIR1 and AFBs are also localized in the nucleus [89]. The Aux/IAA11.1 variant that is localized in the cytoplasm could not be degraded by TIR1, but it also would not be able to repress ARF transcriptional activity. Therefore, the role of this protein variant is currently unclear. Interestingly, we also observed that the intensity of the YFP signal was much higher in YFP::Aux/IAA11.1 than in YFP::Aux/IAA11.2, suggesting that Aux/IAA11.1 may be more stable, presumably because it cannot become a substrate for TIR1 and eventually be degraded.

The results of this study emphasize the importance of Aux/IAA11 in the acclimation to chloroplast-specific stresses and its crosstalk with auxin signaling. In further research, it would be valuable to better understand the molecular function of Aux/IAA11 and to analyze the putative TFs that can regulate the expression of *Aux/IAA11*, and identify Aux/IAA11 protein partners. This should help us determine the exact role of Aux/IAA11 in abiotic stress responses. It was suggested that Aux/IAAs can act as a hub integrating signals from diverse pathways, so determining their exact role might be challenging, however, extremely interesting [21].

## 4. Materials and Methods

### 4.1. Preparation of Plant Material and Growth Conditions

Plant materials were grown in Jiffy pots (Jiffy Products International, The Netherlands) in a growing chamber under ~150 µmol m^−2^ s^−1^ of photons in a long day photoperiod, 16 h of day and 8 h of night, at a temperature of 22 °C. All mutants used in this research were ordered from the NASC. Prior to sowing, seeds were stratified at 4 °C for 48 h in darkness.

### 4.2. Reverse Genetic Screen

T-DNA mutants were genotyped by PCR using the primers listed in Table S2. Screening was performed on homozygous recessive lines, where both alleles of the gene were disrupted by T-DNA insertion. Eight to ten 4–5-week old rosettes from each T-DNA mutant line were used for trials.

For the UV-AB treatment, we used 1000 mJ cm^−2^ of UV-AB, using a UVC 500 Crosslinker (Hoefer Pharmacia Biotech, Holliston, MA, USA). As source of radiation, we used UV-A (TL8WBLB, Philips, The Netherlands) and UV-B lamps (G8T5E, Sankyo Denki, Hiratsuka, Japan). Ion leakage was measured 72 h after stress, with an InoLab Cond Level 1 precision conductivity meter (WTW, Weilheim, Germany), as described previously by Burdiak et al. [90]; except total ion leakage was measured after freezing samples at −80 °C overnight instead of autoclaving them.

For treatments with photosynthesis inhibitors we cut leaf discs out of 4–5-week-old Arabidopsis leaves and put them adaxial side up into 60 μM DCMU solution in darkness and later transferred them to a growing chamber under ~150 µmol m^−2^ s^−1^ of photons. Chlorophyll *a* fluorescence was measured after 0, 1, 2, and 3 h of treatment using a FluorCam 800MF (Photon Systems Instruments, Drásov, Czech Republic), as described previously by Gawroński et al. [91]. Methyl viologen treatment was performed in a similar manner, but we used 5 μM of MV solution and measured chlorophyll *a* fluorescence for up to 4 h (0, 1, 2, 3, 4 h) during the treatment.

### 4.3. RNA Isolation and qRT-PCR Analysis

Whole Arabidopsis rosettes, prior to RNA isolation, were frozen in liquid nitrogen and ground into a powder using a mortar and pestle, not allowing them to thaw in the process. RNA was isolated using a Universal RNA Purification Kit (EurX, Gdańsk, Poland) from 100 mg of tissue, according to manufacturer’s protocol. cDNA were synthetized with a High-Capacity cDNA Reverse Transcription Kit (Applied Biosystems™, San Francisco, CA, USA) using 1000 ng of RNA as a matrix for reverse transcriptase. Then, 10 ng of cDNA was used as matrix for qPCR using iTaq Universal SYBR Green Supermix (Bio-Rad, Hercules, CA, USA). qPCRs were run on a 7500 Fast Real-Time PCR System (Applied Biosystems, Francisco, CA, USA). Primers used in qPCR experiment are listed in Table S2. Amplification efficiency was calculated on the basis of an amplification curve using LinRegPCR 11.0 software [92]. Relative gene expressions were calculated and normalized using the Relative Expression Software Tool (REST 2009) with *UBIQUITIN-PROTEIN LIGASE 7* (*UPL7*) and *YELLOW-LEAF-SPECIFIC GENE 8* (*YLS8*) as references.

To induce high-light intensity stress, plants were illuminated with ~1000 µmol m^−2^ s^−1^ of photons using SL 3500 white LED lamps (Photon Systems Instruments, Drásov, Czech Republic) for up to 72 h in constant light.

### 4.4. UV-AB Treatment of Aux/IAA11 Mutants

Four to five week old Arabidopsis plants were exposed once to 1500 mJ cm^−2^ of UV-AB. Source of UV-AB radiation and ion leakage measurements were made as described for the reverse genetic screen.

### 4.5. DCMU Treatment of Aux/IAA11 Mutants

Leaf discs cut out of 4–5-week-old Arabidopsis leaves were put adaxial side up into 60 μM DCMU solution in darkness and later transferred to a growing chamber under ~150 µmol m^−2^ s^−1^ of photons. Chlorophyll *a* fluorescence was measured after 0, 1, 2, and 3 h of treatment using a FluorCam 800MF (Photon Systems Instruments, Drásov, Czech Republic).

### 4.6. H_2_O_2_ Measurements and SOSG Staining

Leaf discs for ROS content measurements were prepared as described previously, but treated with 200 μM DCMU solution in darkness and later transferred to a growing chamber under ~150 µmol m^−2^ s^−1^ of photons for 15 min. After DCMU treatment, they were rinsed 3× with PBS buffer (EurX, Poland) and stained with SOSG (Invitrogen, Waltham, MA, USA), as described by Flors et al. [56]. Sections for microscopic observations were cut out of middle of the leaf discs after staining. H_2_O_2_ content was measured using an Amplex™ Red Hydrogen Peroxide/Peroxidase Assay Kit (Invitrogen, Waltham, MA, USA), according to manufacturer’s protocol, after crushing whole leaf discs with a polyethylene pestle in 1.5-mL eppendorf type tube with 1x reaction buffer on ice at 4 °C. Absorbance was measured using Multiskan GO (ThermoFisher Scientific, Waltham, MA, USA). Chlorophyll *a* fluorescence was measured after 15 min of DCMU treatment using a FluorCam 800MF (Photon Systems Instruments, Drásov, Czech Republic).

### 4.7. 2,4-Dichlorophenoxyacetic Acid Susceptibility Assay

Autoclave sterilized ½ Murashige and Skoog media with agar (pH 5, 8) were supplemented with filter-sterilized solution of 2,4-D, to a final concentration of 10 nM. Arabidopsis seeds, after surface sterilization with sodium hypochlorite solution, were stratified in 4 °C for 48 h, sown onto plates, and incubated vertically in a growing chamber at 22 °C under a long day photoperiod (16 h of day and 8 h of night, ~150 µmol m^−2^ s^−1^ of photons) for 5–7 days. The placement of seeds from different genotypes was randomized on each plate, to mitigate its effect on observed phenotype. Root lengths were measured using ImageJ software.

### 4.8. Preparation of DNA Constructs for Tobacco and Onion Transformation

The coding site (CDS) of the two *Aux/IAA11* splicing variants, AT4G28640.1 and AT4G28640.2, were amplified by PCR, using the primers listed in Table S2 and Col-0 cDNA as a matrix. After electrophoresis, products with expected length were purified from gel and inserted into a pENTR/D-TOPO vector (ThermoFisher Scientific, Waltham, MA, USA), in order to obtain the entry clones. Subcloning of products into the destination vector pGWB442 [93] was performed using Gateway LR Clonase II enzyme mix (ThermoFisher Scientific, Waltham, MA, USA). The obtained DNA constructs were transformed into *Agrobacterium tumefaciens* (C58C1).

### 4.9. Transient Expression in Nicotiana Benthamiana and Allium Cepa Leaves

*Allium cepa* was transformed as described previously by Xu et al. [94]. *Nicotiana benthamiana* was transformed according to the Joern Klinkenberg protocol [95]. Both experiments were performed using *Agrobacterium tumefaciens* (C58C1) carrying prepared DNA constructs. Fusion proteins, p35S::YFP:IAA11, were visualized using confocal microscopy, 72 h after agroinfiltration. Adaxial epidermis from transformed onions were torn off of the leaf and put into 300 nM DAPI (ThermoFisher Scientific, Waltham, MA, USA) solution in PBS for 20 min and washed 3 times with PBS before visualization. Tobacco observations were carried out on thin sections cut out of a 3rd or 4th agroinfiltrated true leaf.

### 4.10. Confocal Microscopy Observations

Observations were carried out under a Zeiss LSM700 confocal microscope (Zeiss, Oberkochen, Germany) using 20× and 40× EC Plan-Neofluar objectives. A 488-nM laser was used to excite the YFP, DAPI, SOSG, and chlorophyll fluorescence. Pearson correlation coefficient was calculated using the Colocalization Finder plugin for ImageJ.

### 4.11. Statistical Analysis

Statistical analysis was performed using Statistica 12 software [96]. Prior to analyzing the data set using Dunnett’s test, we tested the data for outliers using Grubbs’ test and also a Shapiro–Wilk test, in order to check for a normal distribution. Analysis of qPCR results was performed using the Relative Expression Software Tool (REST 2009) [97]. Asterisks on graphs represent statistical significance levels based on the *p*-value: * *p* < 0.05; ** *p* < 0.01; *** *p* < 0.001.

## Data Availability

Not applicable.

## Supplementary Materials

The following supporting information can be downloaded at: https://www.mdpi.com/article/10.3390/ijms232113386/s1.

## Abbreviations

2,4-D	2,4-Dichlorophenoxyacetic acid
ABRE	Abscisic acid responsive element
AFB	Auxin signaling F-BOX protein
ARF	Auxin responsive factor
AuxRE	Auxin responsive element
*axr2*	*auxin resistant 2*
bZIP	basic leucine zipper transcription factor
cDNA	Complementary DNA
CDS	CDS Coding site
DAPI	4′,6-Diamidino-2-Phenylindole, Dihydrochloride
DCMU	3-(3,4-dichlorophenyl)-1,1-dimethylurea
DREB2A	Dehydration-responsive element-binding protein 2A
GPx	Glutathione peroxidases
IAA	Indole-3-acetic acid
LOOH	Lipid hydroperoxide
LRE	Light responsive element
MV	Methyl viologen
NAA	1-napthaleneacetic acid
NASC	Nottingham Arabidopsis Stock Centre
NGE	Nuclear gene expression
PAA	Phenylacetic acid
PCD	Programmed cell death
PIN	Pin-formed proteins
ROS	Reactive Oxygen Species
SOSG	Singlet Oxygen Sensor Green
TIR1	Transport Inhibitor Response 1
qPCR	Quantitative PCR

## References

[1] Wang Y., Selinski J., Mao C., Zhu Y., Berkowitz O., Whelan J. (2020). Linking Mitochondrial and Chloroplast Retrograde Signalling in Plants. Philos. Trans. R. Soc. B Biol. Sci..

[2] Mielecki J., Gawroński P., Karpiński S. (2020). Retrograde Signaling: Understanding the Communication between Organelles. Int. J. Mol. Sci..

[3] Wu G.Z., Bock R. (2021). GUN Control in Retrograde Signaling: How GENOMES UNCOUPLED Proteins Adjust Nuclear Gene Expression to Plastid Biogenesis. Plant Cell.

[4] Jiang J., Dehesh K. (2021). Plastidial Retrograde Modulation of Light and Hormonal Signaling: An Odyssey. New Phytol..

[5] Morffy N., Strader L.C. (2020). Old Town Roads: Routes of Auxin Biosynthesis across Kingdoms. Curr. Opin. Plant Biol..

[6] Leyser O. (2018). Auxin Signaling. Plant Physiol.

[7] Damodaran S., Strader L.C. (2019). Indole 3-Butyric Acid Metabolism and Transport in Arabidopsis Thaliana. Front. Plant Sci..

[8] Du M., Spalding E.P., Gray W.M. (2020). Rapid Auxin-Mediated Cell Expansion. Annu. Rev. Plant Biol..

[9] Sańko-Sawczenko I., Łotocka B., Czarnocka W. (2016). Expression Analysis of PIN Genes in Root Tips and Nodules of Medicago Truncatula. Int. J. Mol. Sci..

[10] Zhou J.J., Luo J. (2018). The PIN-FORMED Auxin Efflux Carriers in Plants. Int. J. Mol. Sci..

[11] Strader L.C., Wheeler D.L., Christensen S.E., Berens J.C., Cohen J.D., Rampey R.A., Bartel B. (2011). Multiple Facets of *Arabidopsis* Seedling Development Require Indole-3-Butyric Acid–Derived Auxin. Plant Cell.

[12] Luo J., Zhou J.J., Zhang J.Z. (2018). Aux/IAA Gene Family in Plants: Molecular Structure, Regulation, and Function. Int. J. Mol. Sci..

[13] Abel S., Oeller P.W., Theologis A. (1994). Early Auxin-Induced Genes Encode Short-Lived Nuclear Proteins. Proc. Natl. Acad. Sci. USA.

[14] Lavy M., Estelle M. (2016). Mechanisms of Auxin Signaling. Development.

[15] Calderón Villalobos L.I.A., Lee S., de Oliveira C., Ivetac A., Brandt W., Armitage L., Sheard L.B., Tan X., Parry G., Mao H. (2012). A Combinatorial TIR1/AFB-Aux/IAA Co-Receptor System for Differential Sensing of Auxin. Nat. Chem. Biol..

[16] Iglesias M.J., Terrile M.C., Correa-Aragunde N., Colman S.L., Izquierdo-Álvarez A., Fiol D.F., París R., Sánchez-López N., Marina A., Calderón Villalobos L.I.A. (2018). Regulation of SCFTIR1/AFBs E3 Ligase Assembly by S-Nitrosylation of Arabidopsis SKP1-Like1 Impacts on Auxin Signaling. Redox Biol..

[17] Szemenyei H., Hannon M., Long J.A. (2008). TOPLESS Mediates Auxin-Dependent Transcriptional Repression during Arabidopsis Embryogenesis. Science.

[18] Kagale S., Rozwadowski K. (2011). EAR Motif-Mediated Transcriptional Repression in Plants: An Underlying Mechanism for Epigenetic Regulation of Gene Expression. Epigenetics.

[19] Niemeyer M., Moreno Castillo E., Ihling C.H., Iacobucci C., Wilde V., Hellmuth A., Hoehenwarter W., Samodelov S.L., Zurbriggen M.D., Kastritis P.L. (2020). Flexibility of Intrinsically Disordered Degrons in AUX/IAA Proteins Reinforces Auxin Co-Receptor Assemblies. Nat. Commun..

[20] Winkler M., Niemeyer M., Hellmuth A., Janitza P., Christ G., Samodelov S.L., Wilde V., Majovsky P., Trujillo M., Zurbriggen M.D. (2017). Variation in Auxin Sensing Guides AUX/IAA Transcriptional Repressor Ubiquitylation and Destruction. Nat. Commun..

[21] Shani E., Salehin M., Zhang Y., Sanchez S.E., Doherty C., Wang R., Mangado C.C., Song L., Tal I., Pisanty O. (2017). Plant Stress Tolerance Requires Auxin-Sensitive Aux/IAA Transcriptional Repressors. Curr. Biol..

[22] Salehin M., Li B., Tang M., Katz E., Song L., Ecker J.R., Kliebenstein D.J., Estelle M. (2019). Auxin-Sensitive Aux/IAA Proteins Mediate Drought Tolerance in Arabidopsis by Regulating Glucosinolate Levels. Nat. Commun..

[23] Wang F., Niu H., Xin D., Long Y., Wang G., Liu Z., Li G., Zhang F., Qi M., Ye Y. (2021). OsIAA18, an Aux/IAA Transcription Factor Gene, Is Involved in Salt and Drought Tolerance in Rice. Front. Plant Sci..

[24] Aslam M., Sugita K., Qin Y., Rahman A. (2020). Aux/IAA14 Regulates MicroRNA-Mediated Cold Stress Response in Arabidopsis Roots. Int. J. Mol. Sci..

[25] Shi H., Liu W., Wei Y., Ye T. (2016). Integration of Auxin/Indole-3-Acetic Acid 17 and RGA-LIKE3 Confers Salt Stress Resistance through Stabilization by Nitric Oxide in Arabidopsis. J. Exp. Bot..

[26] Hou Y., Li H., Zhai L., Xie X., Li X., Bian S. (2020). Identification and Functional Characterization of the Aux/IAA Gene VcIAA27 in Blueberry. Plant Signal Behav..

[27] Sato A., Yamamoto K.T. (2008). Overexpression of the Non-Canonical Aux/IAA Genes Causes Auxin-Related Aberrant Phenotypes in Arabidopsis. Physiol. Plant.

[28] Nagpal P., Walker L.M., Young J.C., Sonawala A., Timpte C., Estelle M., Reed J.W. (2000). AXR2 Encodes a Member of the Aux/IAA Protein Family. Plant Physiol..

[29] Yang X., Lee S., So J., Dharmasiri S., Dharmasiri N., Ge L., Jensen C., Hangarter R., Hobbie L., Estelle M. (2004). The IAA1 Protein Is Encoded by AXR5 and Is a Substrate of SCFTIR1. Plant J..

[30] Zhang H., Tan X., Li L., He Y., Hong G., Li J., Lin L., Cheng Y., Yan F., Chen J. (2019). Suppression of Auxin Signalling Promotes Rice Susceptibility to Rice Black Streaked Dwarf Virus Infection. Mol. Plant Pathol..

[31] Zhang A., Yang X., Lu J., Song F., Sun J., Wang C., Lian J., Zhao L., Zhao B. (2021). OsIAA20, an Aux/IAA Protein, Mediates Abiotic Stress Tolerance in Rice through an ABA Pathway. Plant Sci..

[32] Liu K., Yuan C., Feng S., Zhong S., Li H., Zhong J., Shen C., Liu J. (2017). Genome-Wide Analysis and Characterization of Aux/IAA Family Genes Related to Fruit Ripening in Papaya (*Carica papaya* L.). BMC Genom..

[33] Watanabe E., Mano S., Hara-Nishimura I., Nishimura M., Yamada K. (2017). HSP90 Stabilizes Auxin Receptor TIR1 and Ensures Plasticity of Auxin Responses. Plant Signal Behav..

[34] Xi Y., Yang Y., Yang J., Zhang X., Pan Y., Guo H. (2021). IAA3-Mediated Repression of PIF Proteins Coordinates Light and Auxin Signaling in Arabidopsis. PLoS Genet..

[35] Xu F., He S., Zhang J., Mao Z., Wang W., Li T., Hua J., Du S., Xu P., Li L. (2018). Photoactivated CRY1 and PhyB Interact Directly with AUX/IAA Proteins to Inhibit Auxin Signaling in Arabidopsis. Mol. Plant.

[36] Jiang J., Xiao Y., Chen H., Hu W., Zeng L., Ke H., Ditengou F.A., Devisetty U., Palme K., Maloof J. (2020). Retrograde Induction of PhyB Orchestrates Ethylene-Auxin Hierarchy to Regulate Growth. Plant Physiol..

[37] Jiang J., Rodriguez-Furlan C., Wang J.Z., de Souza A., Ke H., Pasternak T., Lasok H., Ditengou F.A., Palme K., Dehesh K. (2018). Interplay of the Two Ancient Metabolites Auxin and MEcPP Regulates Adaptive Growth. Nat Commun.

[38] Phua S.Y., Yan D., Chan K.X., Estavillo G.M., Nambara E., Pogson B.J. (2018). The Arabidopsis SAL1-PAP Pathway: A Case Study for Integrating Chloroplast Retrograde, Light and Hormonal Signaling in Modulating Plant Growth and Development?. Front. Plant Sci..

[39] Ishiga Y., Watanabe M., Ishiga T., Tohge T., Matsuura T., Ikeda Y., Hoefgen R., Fernie A.R., Mysore K.S. (2017). The SAL-PAP Chloroplast Retrograde Pathway Contributes to Plant Immunity by Regulating Glucosinolate Pathway and Phytohormone Signaling. Mol. Plant Microbe Interact..

[40] Ivanova A., Law S.R., Narsai R., Duncan O., Lee J.H., Zhang B., van Aken O., Radomiljac J.D., van der Merwe M., Yi K.K. (2014). A Functional Antagonistic Relationship between Auxin and Mitochondrial Retrograde Signaling Regulates Alternative Oxidase1a Expression in Arabidopsis. Plant Physiol..

[41] He C., Liew L.C., Yin L., Lewsey M.G., Whelan J., Berkowitz O. (2022). The Retrograde Signaling Regulator ANAC017 Recruits the MKK9–MPK3/6, Ethylene, and Auxin Signaling Pathways to Balance Mitochondrial Dysfunction with Growth. Plant Cell.

[42] Kerchev P.I., de Clercq I., Denecker J., Mühlenbock P., Kumpf R., Nguyen L., Audenaert D., Dejonghe W., van Breusegem F. (2014). Mitochondrial Perturbation Negatively Affects Auxin Signaling. Mol. Plant.

[43] Tivendale N.D., Millar A.H. (2022). How Is Auxin Linked with Cellular Energy Pathways to Promote Growth?. New Phytol..

[44] Gläßer C., Haberer G., Finkemeier I., Pfannschmidt T., Kleine T., Leister D., Dietz K.J., Häusler R.E., Grimm B., Mayer K.F.X. (2014). Meta-Analysis of Retrograde Signaling in Arabidopsis Thaliana Reveals a Core Module of Genes Embedded in Complex Cellular Signaling Networks. Mol. Plant.

[45] Gawroński P., Burdiak P., Scharff L.B., Mielecki J., Górecka M., Zaborowska M., Leister D., Waszczak C., Karpiński S. (2021). CIA2 and CIA2-LIKE Are Required for Optimal Photosynthesis and Stress Responses in *Arabidopsis thaliana*. Plant J..

[46] ARAMEMNON—Plant Membrane Protein Database (Release 8.1). http://aramemnon.uni-koeln.de/index.ep.

[47] Schwacke R., Schneider A., van der Graaff E., Fischer K., Catoni E., Desimone M., Frommer W.B., Flügge U.I., Kunze R. (2003). ARAMEMNON, a Novel Database for Arabidopsis Integral Membrane Proteins. Plant Physiol..

[48] Hruz T., Laule O., Szabo G., Wessendorp F., Bleuler S., Oertle L., Widmayer P., Gruissem W., Zimmermann P. (2008). Genevestigator V3: A Reference Expression Database for the Meta-Analysis of Transcriptomes. Adv. Bioinform..

[49] Scholl R.L., May S.T., Ware D.H. (2000). Seed and Molecular Resources for Arabidopsis. Plant Physiol..

[50] Sun X., Feng P., Xu X., Guo H., Ma J., Chi W., Lin R., Lu C., Zhang L. (2011). A Chloroplast Envelope-Bound PHD Transcription Factor Mediates Chloroplast Signals to the Nucleus. Nat. Commun..

[51] Chen Y., Li T., Yang Q., Zhang Y., Zou J., Bian Z., Wen X. (2019). UVA Radiation Is Beneficial for Yield and Quality of Indoor Cultivated Lettuce. Front. Plant Sci..

[52] Britt A.B. (1995). Repair of DNA Damage Induced by Ultraviolet Radiation. Plant Physiol..

[53] Shi C., Liu H. (2021). How Plants Protect Themselves from Ultraviolet-B Radiation Stress. Plant Physiol..

[54] Sztatelman O., Grzyb J., Gabryś H., Banaś A.K. (2015). The Effect of UV-B on Arabidopsis Leaves Depends on Light Conditions after Treatment. BMC Plant Biol..

[55] Wituszyńska W., Szechyńska-Hebda M., Sobczak M., Rusaczonek A., Kozlowska-Makulska A., Witoń D., Karpiński S. (2015). LESION SIMULATING DISEASE 1 And ENHANCED DISEASE SUSCEPTIBILITY 1 Differentially Regulate UV-C-Induced Photooxidative Stress Signalling and Programmed Cell Death in Arabidopsis Thaliana. Plant Cell Env..

[56] Flors C., Fryer M.J., Waring J., Reeder B., Bechtold U., Mullineaux P.M., Nonell S., Wilson M.T., Baker N.R. (2006). Imaging the Production of Singlet Oxygen in Vivo Using a New Fluorescent Sensor, Singlet Oxygen Sensor Green. J. Exp. Bot..

[57] Karpinski S., Reynolds H., Karpinska B., Wingsle G., Creissen G., Mullineaux P. (1999). Systemic Signaling and Acclimation in Response to Excess Excitation Energy in Arabidopsis. Science.

[58] Fufezan C., Rutherford A.W., Krieger-Liszkay A. (2002). Singlet Oxygen Production in Herbicide-Treated Photosystem II. FEBS Lett..

[59] Cui F., Brosché M., Shapiguzov A., He X.Q., Vainonen J.P., Leppälä J., Trotta A., Kangasjärvi S., Salojärvi J., Kangasjärvi J. (2019). Interaction of Methyl Viologen-Induced Chloroplast and Mitochondrial Signalling in Arabidopsis. Free Radic. Biol. Med..

[60] Tamura K., Stecher G., Kumar S. (2021). MEGA11: Molecular Evolutionary Genetics Analysis Version 11. Mol. Biol. Evol..

[61] Tiwari S.B., Hagen G., Guilfoyle T. (2003). The Roles of Auxin Response Factor Domains in Auxin-Responsive Transcription. Plant Cell.

[62] Freire-Rios A., Tanaka K., Crespo I., van der Wijk E., Sizentsova Y., Levitsky V., Lindhoud S., Fontana M., Hohlbein J., Roeland Boer D. (2020). Architecture of DNA Elements Mediating ARF Transcription Factor Binding and Auxin-Responsive Gene Expression in Arabidopsis. Proc. Natl. Acad. Sci. USA.

[63] Sun X.L., Li Y., Cai H., Bai X., Ji W., Ji Z.J., Zhu Y.M. (2011). Arabidopsis BZIP1 Transcription Factor Binding to ABRE Cis-Element Regulates Abscisic Acid Signal Transduction. Acta Agron. Sin..

[64] Tzvetkova-Chevolleau T., Franck F., Alawady A.E., Dall’Osto L., Carrière F., Bassi R., Grimm B., Nussaume L., Havaux M. (2007). The Light Stress-Induced Protein ELIP2 Is a Regulator of Chlorophyll Synthesis in Arabidopsis Thaliana. Plant J..

[65] Czarnocka W., Karpiński S. (2018). Friend or Foe? Reactive Oxygen Species Production, Scavenging and Signaling in Plant Response to Environmental Stresses. Free Radic. Biol. Med..

[66] Miyamoto S., Ronsein G.E., Prado F.M., Uemi M., Corrêa T.C., Toma I.N., Bertolucci A., Oliveira M.C.B., Motta F.D., Medeiros M.H.G. (2007). Biological Hydroperoxides and Singlet Molecular Oxygen Generation. IUBMB Life.

[67] Mai Y.-X., Wang L., Yang H.-Q. (2011). A Gain-of-Function Mutation in IAA7/AXR2 Confers Late Flowering under Short-Day Light in ArabidopsisF. J. Integr. Plant Biol..

[68] Lanctot A., Taylor-Teeples M., Oki E.A., Nemhauser J.L. (2020). Specificity in Auxin Responses Is Not Explained by the Promoter Preferences of Activator ARFs. Plant Physiol..

[69] Kim J.-S., Mizoi J., Yoshida T., Fujita Y., Nakajima J., Ohori T., Todaka D., Nakashima K., Hirayama T., Shinozaki K. (2011). An ABRE Promoter Sequence Is Involved in Osmotic Stress-Responsive Expression of the DREB2A Gene, Which Encodes a Transcription Factor Regulating Drought-Inducible Genes in Arabidopsis. Plant Cell Physiol..

[70] Geisler M., Kleczkowski L.A., Karpinski S. (2006). A Universal Algorithm for Genome-Wide in Silicio Identification of Biologically Significant Gene Promoter Putative *Cis* -Regulatory-Elements; Identification of New Elements for Reactive Oxygen Species and Sucrose Signaling in Arabidopsis. Plant J..

[71] Li M., Kim C. (2022). Chloroplast ROS and Stress Signaling. Plant Commun..

[72] Galvez-Valdivieso G., Fryer M.J., Lawson T., Slattery K., Truman W., Smirnoff N., Asami T., Davies W.J., Jones A.M., Baker N.R. (2009). The High Light Response in Arabidopsis Involves ABA Signaling between Vascular and Bundle Sheath CellsW. Plant Cell.

[73] Pornsiriwong W., Estavillo G.M., Chan K.X., Tee E.E., Ganguly D., Crisp P.A., Phua S.Y., Zhao C., Qiu J., Park J. (2017). A Chloroplast Retrograde Signal, 3’phosphoadenosine 5′-Phosphate, Acts as a Secondary Messenger in Abscisic Acid Signaling in Stomatal Closure and Germination. Elife.

[74] Fryer M.J., Ball L., Oxborough K., Karpinski S., Mullineaux P.M., Baker N.R. (2003). Control of *Ascorbate Peroxidase 2* Expression by Hydrogen Peroxide and Leaf Water Status during Excess Light Stress Reveals a Functional Organisation of *Arabidopsis* Leaves. Plant J..

[75] Karpinski S., Escobar C., Karpinska B., Creissen G., Mullineaux P.M. (1997). Photosynthetic Electron Transport Regulates the Expression of Cytosolic Ascorbate Peroxidase Genes in Arabidopsis during Excess Light Stress. Plant Cell.

[76] Huang J., Zhao X., Chory J. (2019). The Arabidopsis Transcriptome Responds Specifically and Dynamically to High Light Stress. Cell Rep..

[77] Yang Y., Zhang L., Chen P., Liang T., Li X., Liu H. (2020). UV-B Photoreceptor UVR8 Interacts with MYB73/MYB77 to Regulate Auxin Responses and Lateral Root Development. EMBO J..

[78] Zhao Y., Xing L., Wang X., Hou Y.J., Gao J., Wang P., Duan C.G., Zhu X., Zhu J.K. (2014). The ABA Receptor PYL8 Promotes Lateral Root Growth by Enhancing MYB77-Dependent Transcription of Auxin-Responsive Genes. Sci. Signal.

[79] Shin R., Burch A.Y., Huppert K.A., Tiwari S.B., Murphy A.S., Guilfoyle T.J., Schachtman D.P. (2007). The Arabidopsis Transcription Factor MYB77 Modulates Auxin Signal Transduction. Plant Cell.

[80] Wan J., Zhang P., Wang R., Sun L., Wang W., Zhou H., Xu J. (2018). UV-B Radiation Induces Root Bending Through the Flavonoid-Mediated Auxin Pathway in Arabidopsis. Front. Plant Sci..

[81] Ahammed G.J., Yu J.Q. (2016). Plant Hormones under Challenging Environmental Factors.

[82] Wituszynska W., Karpinski S. (2013). Programmed Cell Death as a Response to High Light, UV and Drought Stress in Plants. Abiotic Stress—Plant Responses and Applications in Agriculture.

[83] Wituszyńska W., Ślesak I., Vanderauwera S., Szechyńska-Hebda M., Kornaś A., van der Kelen K., Mühlenbock P., Karpińska B., Maćkowski S., van Breusegem F. (2013). Lesion simulating disease, enhanced disease susceptibility, and phytoalexin deficient conditionally regulate cellular signaling homeostasis, photosynthesis, water use efficiency, and seed yield in Arabidopsis. Plant Physiol..

[84] Biever J.J., Brinkman D., Gardner G. (2014). UV-B Inhibition of Hypocotyl Growth in Etiolated Arabidopsis Thaliana Seedlings Is a Consequence of Cell Cycle Arrest Initiated by Photodimer Accumulation. J. Exp. Bot..

[85] Huq E. (2018). Direct Convergence of Light and Auxin Signaling Pathways in Arabidopsis. Mol. Plant.

[86] Gören-Sağlam N., Harrison E., Breeze E., Öz G., Buchanan-Wollaston V. (2020). Analysis of the Impact of Indole-3-Acetic Acid (IAA) on Gene Expression during Leaf Senescence in Arabidopsis Thaliana. Physiol. Mol. Biol. Plants.

[87] Zhao Y., Mou M., Li P., Huang Y., Zhai X., Ma Y., Liu J., Yu X. (2015). Theoretical Modeling of the Aux/IAA Negative Feedback Circuit in Plants. S. Afr. J. Bot..

[88] Lv B., Yu Q., Liu J., Wen X., Yan Z., Hu K., Li H., Kong X., Li C., Tian H. (2020). Non-canonical AUX / IAA Protein IAA 33 Competes with Canonical AUX / IAA Repressor IAA 5 to Negatively Regulate Auxin Signaling. EMBO J.

[89] Dharmasiri N., Dharmasiri S., Weijers D., Lechner E., Yamada M., Hobbie L., Ehrismann J.S., Jürgens G., Estelle M. (2005). Plant Development Is Regulated by a Family of Auxin Receptor F Box Proteins. Dev. Cell.

[90] Burdiak P., Rusaczonek A., Witoń D., Głów D., Karpiński S. (2015). Cysteine-Rich Receptor-like Kinase CRK5 as a Regulator of Growth, Development, and Ultraviolet Radiation Responses in Arabidopsis Thaliana. J. Exp. Bot..

[91] Gawroński P., Witoń D., Vashutina K., Bederska M., Betliński B., Rusaczonek A., Karpiński S. (2014). Mitogen-Activated Protein Kinase 4 Is a Salicylic Acid-Independent Regulator of Growth but Not of Photosynthesis in Arabidopsis. Mol. Plant.

[92] Ramakers C., Ruijter J.M., Lekanne Deprez R.H., Moorman A.F.M. (2003). Assumption-Free Analysis of Quantitative Real-Time Polymerase Chain Reaction (PCR) Data. Neurosci. Lett..

[93] Nakagawa T., Kurose T., Hino T., Tanaka K., Kawamukai M., Niwa Y., Toyooka K., Matsuoka K., Jinbo T., Kimura T. (2007). Development of Series of Gateway Binary Vectors, PGWBs, for Realizing Efficient Construction of Fusion Genes for Plant Transformation. J. Biosci. Bioeng..

[94] Xu K., Huang X., Wu M., Wang Y., Chang Y., Liu K., Zhang J., Zhang Y., Zhang F., Yi L. (2014). A Rapid, Highly Efficient and Economical Method of Agrobacterium-Mediated In Planta Transient Transformation in Living Onion Epidermis. PLoS ONE.

[95] Klinkenberg J. (2014). Extraction of Chloroplast Proteins from Transiently Transformed Nicotiana Benthamiana Leaves. Bio. Protoc..

[96] Statistica—StatSoft Poland. https://www.statsoft.pl/Pelna-lista-programow-Statistica/.

[97] Pfaffl M.W., Horgan G.W., Dempfle L. (2002). Relative Expression Software Tool (REST) for Group-Wise Comparison and Statistical Analysis of Relative Expression Results in Real-Time PCR. Nucleic Acids Res..

