# Supplementary material for: Aux/IAA11 Is Required for UV-AB Tolerance and Auxin Sensing in Arabidopsis thaliana
Source: Int J Mol Sci. 2022 Nov 2;23(21):13386. doi: 10.3390/ijms232113386 (PMC9655273; doi:10.3390/ijms232113386)
Supplement: Supplementary file 1 [file ijms-23-13386-s001.zip › ijms-2000444-supplementary.pdf]

Table S1. Mutants used in reverse genetic screen.

TF family	AGI code	Size (aa)	mutant	mutation location	Comments	UV-AB susceptibility	DCMU susceptibility	MV susceptibility
AUX/IAA	AT4G28640	302	GK-685A08	intron	IAA11	YES	YES	NO
bHLH	AT5G57150	226	SALK_016841	intron	BHLH35	NO	NO	NO
bZIP	AT4G37730	305	GK-031D04	exon	bZIP7	NO	NO	NO
NAC	AT1G33060	648	SALK_031642	exon	NAC014	NO	NO	NO
WRKY	AT2G03340	513	SALK_119051	exon	WRKY3	NO	NO	NO
ZF	AT2G29660	373	SALK_119814	exon	ZF	NO	NO	NO
ZF	AT3G47550	249	SALK_090823	exon	F1P2.100	YES	NO	NO
ZF	AT5G05660	880	SALK_140301	exon	NFXL2	YES	NO	NO
ZF	AT5G40710	272	SALK_087836	exon	MNF13.27	NO	NO	NO
ZF	AT5G40710	272	WiscDsLox407C08	exon	MNF13.27	NO	NO	NO

Table S2. List of primers used in this study.

Gene name	AGI code	Forward primer sequence	Reverse primer sequence	Used for
AUX/IAA11	AT4G28640	TTCAGGGCCTTGTAAAATCAG	ACCACGGATACTACCGTTTTG	SALKseq_033787 genotyping
AUX/IAA11	AT4G28640	ATGTTTTCATTATCCGGAGCC	ATTCCATGGCTGCAACTAGTG	SALKseq_115462 genotyping
AUX/IAA11	AT4G28640	ATGTTTTCATTATCCGGAGCC	ATTCCATGGCTGCAACTAGTG	SAILseq_567_H06 genotyping
AUX/IAA11	AT4G28640	ATGTTTTCATTATCCGGAGCC	CAGAGCTAGCGTAATTGCTGG	GK-685A08 genotyping
AUX/IAA11	AT4G28640	GAGCTAGCGTAATTGCTGGG	TGTTCATTCTGTAAGTCCTT	qPCR, intron flanking
AUX/IAA11	AT4G28640	CAGATGGGTCTTCCGGATTA	CATCTGAGCTTTACCAGTAG	qPCR, *IAA11.1* splicing variant
AUX/IAA11	AT4G28640	CAGATGGGTCTTCCGGATTA	CATCTTTACCAGTAGCCTCC	qPCR, *IAA11.2* splicing variant
AUX/IAA10	AT1G04100	GCTGATTCTTCTCCGGCTGC	CCGCCTTCCGTAGCTAAAGA	qPCR
AUX/IAA12	AT1G04550	TGAATCCGAAAGTTCAGGGC	CAAGTTTTCGTAAGACGAAT	qPCR
AUX/IAA13	AT2G33310	TCAAGTTGTTGGATGGCCTC	TTTCTTCTTACCAGCTTCTT	qPCR
		TGATCCATGTAGATTTCCCGGAC ATGAAG		T-DNA binding, genotyping of Wisc mutants (WiscDsLoxHS_L4)
		ATTTTGCCGATTTCGGAAC		T-DNA binding, genotyping of SALK mutants (LBb1_3)
		TAGCATCTGAATTTCATAAC-CAATCTCGATACAC		T-DNA binding, genotyping of SAIL mutants (LB3)
		ATAATAACGCTGCG-GACATCTACATTTT		T-DNA binding, genotyping of GABI-kat mutants (o8474)
bHLH35	AT5G57150	TGGACCAAATTACAATAATT-GTGG	CTCACCTCTTTCTCTGGCATG	SALK_016841 genotyping
bZIP7	AT4G37730	CTCTCCAGTGTGTGTCCATCC	AATTGTTTTGTGTCGTCCGAG	GK-031D04 genotyping
AXX17	AT2G13960	TTGCTCGTCTCATAGGTCCAC	AGTCGGTTTTGAATAAACCGG	SALK_009652 genotyping
NAC014	AT1G33060	AGTTGTGAGGTCTTTGCTTGC	AAACGTTGTTATGAAGTGGCG	SALK_031642 genotyping
WRKY3	AT2G03340	GCAAC-CAATGCAAATTTAAATTC	CAGCAAAATGGTTTGGATTTG	SALK_119051 genotyping
ZF	AT2G29660	GAATTTCGGCTCCAAAGTTTC	TAAGATTCGATTGATCACCGG	SALK_119814 genotyping
F1P2.100	AT3G47550	GTCTGCTTCCAAGAAATGACG	GGTTCCAGATTTCGAAAAAGC	SALK_090823 genotyping
NFXL2	AT5G05660	GCATTGTTCCGATAGCTTCTG	TTCATCCAATCTGAAAGCCAC	SALK_140301 genotyping
MNF13.27	AT5G40710	TAATACCGTGAAGACGGTTGG	GGGATTCTCACTTCCCACTTC	SALK_087836 genotyping
MNF13.27	AT5G40710	GACGCAAGAAGAAATCTGCAG	GGGATTCTCACTTCCCACTTC	WiscDsLox407C08 genotyping
AUX/IAA11	AT4G28640	CACCATGGAAGGCGGTTCC	GGATCCTCATAATATCATCT	IAA11.1 subcellular localization
AUX/IAA11	AT4G28640	CACCATGGAAGGCGGTTCC	GGATCCTTACAAAGAGAACA	IAA11.2 subcellular localization

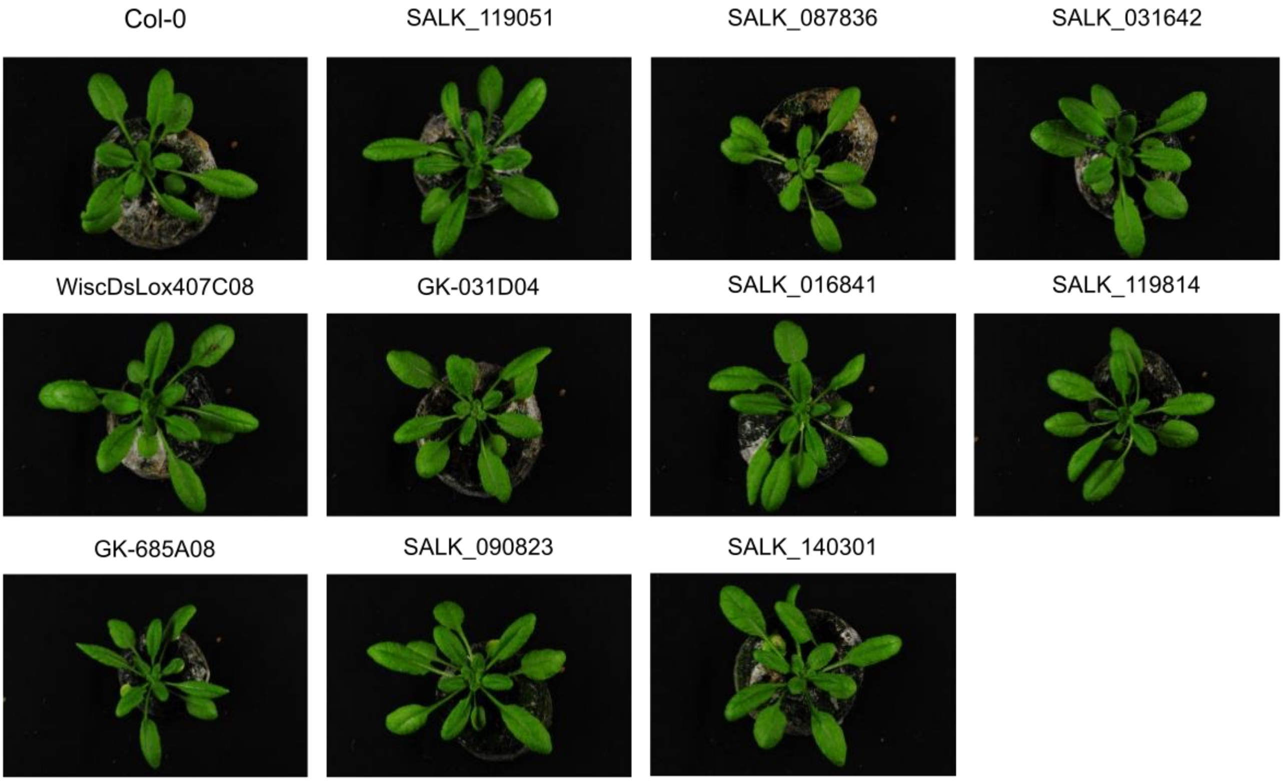

Figure S1. Phenotypes of mutants used in reverse genetic screen. Four week old rosettes of selected mutant lines did not exhibit abnormal phenotype when compared to the wild-type.

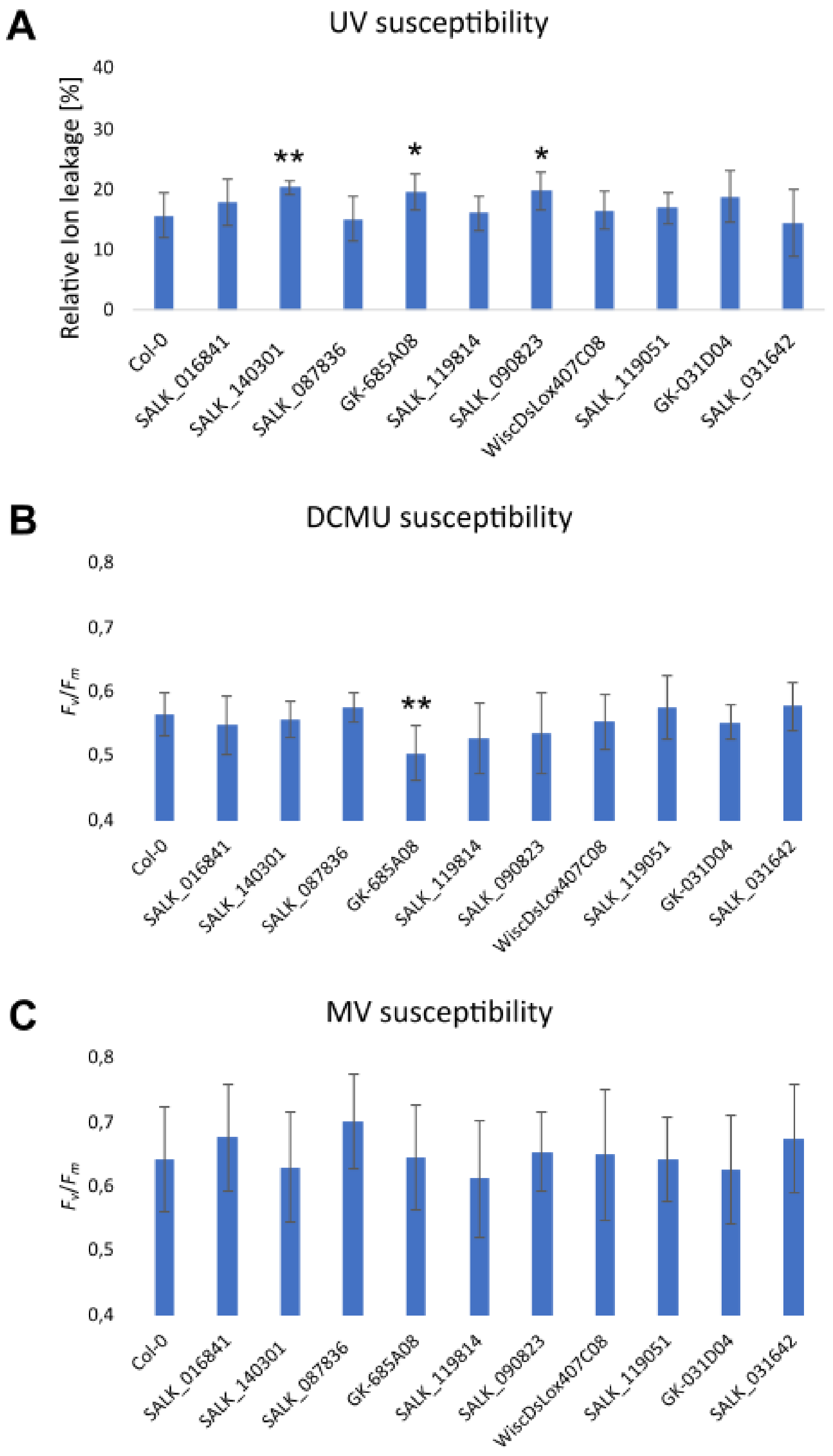

Figure S2. Reverse genetic screen results. (**A**) Relative ion leakage measured 72 hours after exposure to 1000 mJ cm^{-2} of UV-AB, n = 8. (**B**) Maximum quantum yield of photosystem II (F_v/F_m) after 3 hours of 60 µM DCMU treatment, n ≥ 8. (**C**) Maximum quantum yield of photosystem II (F_v/F_m) after 4 hours of 5 µM MV treatment. n ≥ 8, t-test (p* < 0,05; p** < 0,01; p*** < 0,001). Error bars represent standard deviation.

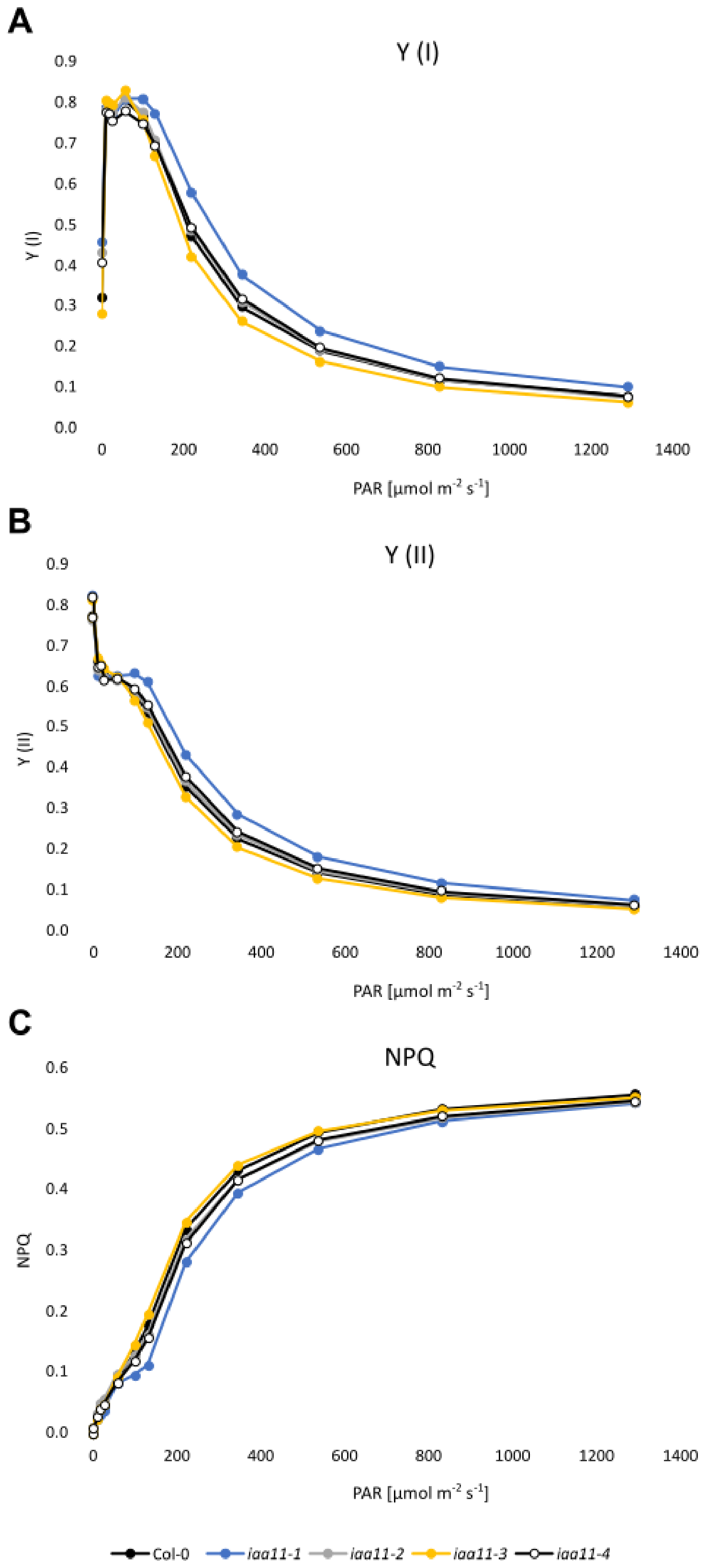

Figure S3. Efficiency of photochemistry and heat dissipation of *Aux/IAA11* mutants. (**A**) Yield of photosystem I. (**B**) Yield of photosystem II. (**C**) Non-photochemical quenching (NPQ) n = 10.

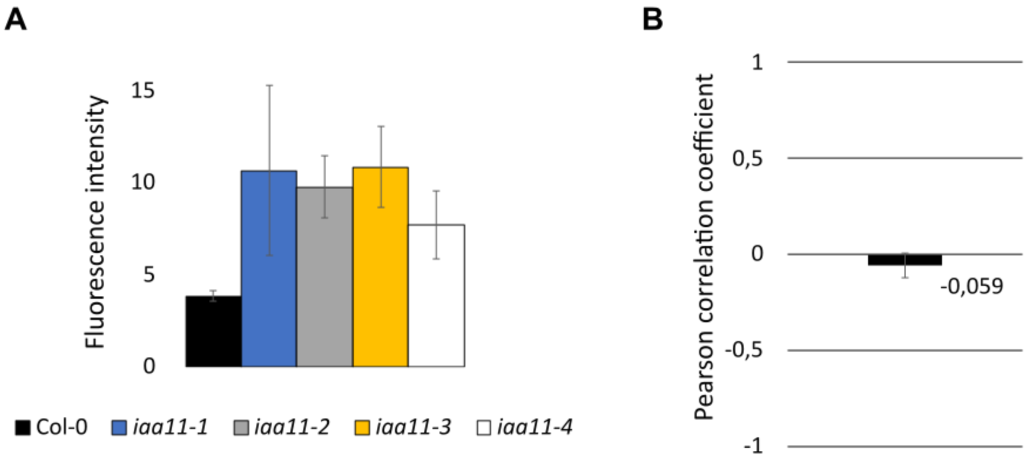

Figure S4. Quantification of SOSG fluorescence intensity. (**A**) Green channel intensity measured with ImageJ software, n ≥ 3. (**B**) Pearson correlation coefficient between SOSG and Chlorophyll *a* fluorescence channels, n = 12. Error bars represent standard deviation.

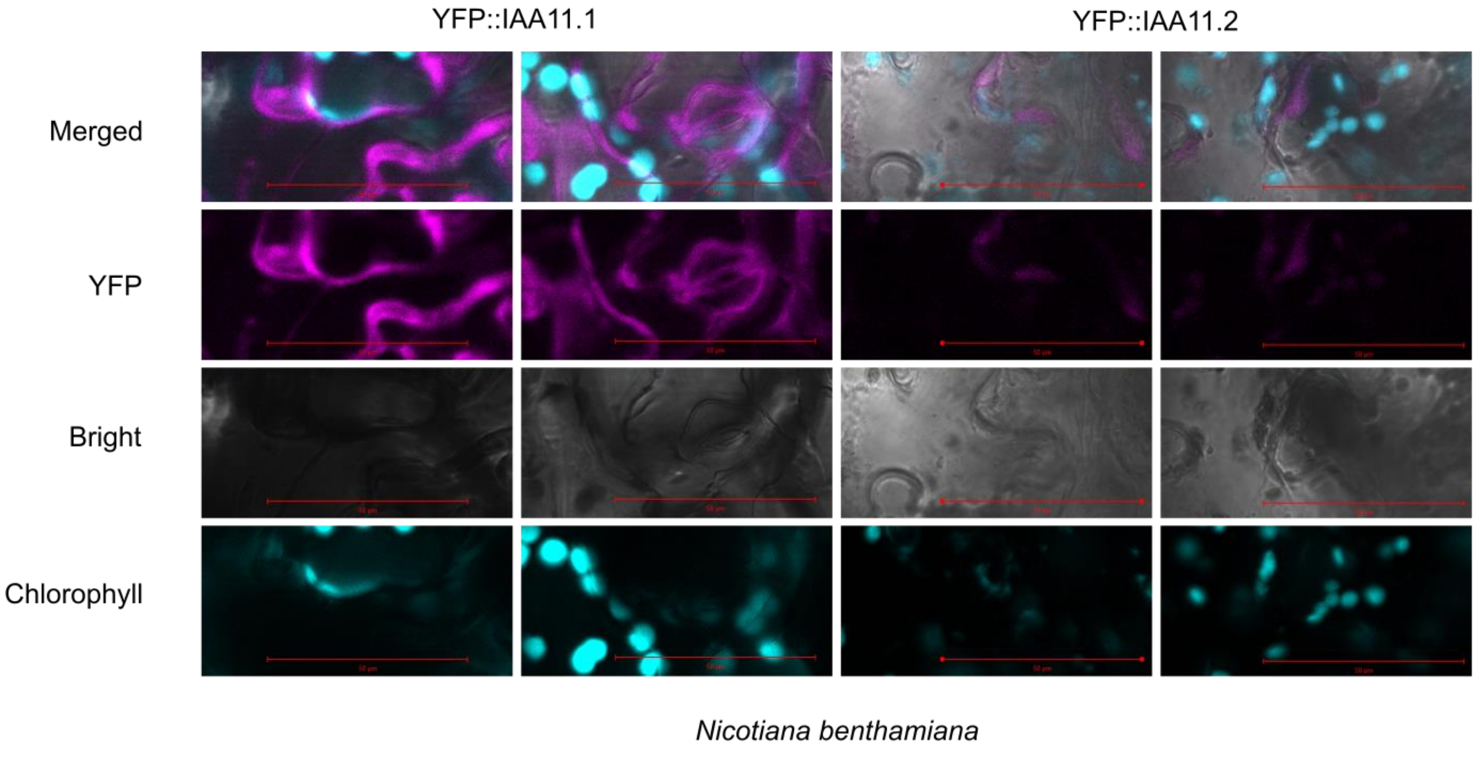

Figure S5. Subcellular localization of IAA11 protein variants in *Nicotiana benthamiana*. Transient expression of fusion proteins: YFP::IAA11.1 and YFP:IAA11.2 in *Nicotiana benthamiana* abaxial epidermis under 35S constitutive promoter. Pictures were taken 72 hours post agrobacterium infiltration using confocal microscopy. Magenta and cyan colors represent YFP and chlorophyll fluorescence respectively. Red scale bars represent 50 µm.

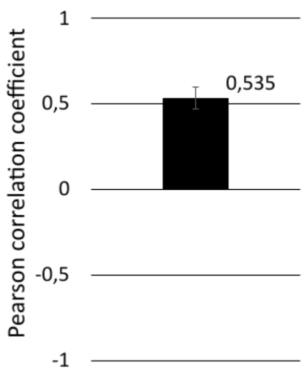

Figure S6. Pearson correlation coefficient between YFP and DAPI channels measured for signals obtained from IAA11.2 protein variant in *Allium cepa*, n = 4. Error bar represent standard deviation.
